# The Implementation of a Business Process Model and Notation for Modeling Patient Health Care Trajectories: Systematic Review

**DOI:** 10.2196/78506

**Published:** 2026-06-09

**Authors:** Jean-Baptiste Gartner, Paolo Landa, Matthew T Haren, Célia Lemaire, Elena Tanfani, Catherine Paquet, Frédéric Bergeron, André Côté

**Affiliations:** 1 Department of Management, Faculty of Administrative Sciences, Laval University, 2325 rue de la Terrasse, Québec, QC, G1V 0A6, Canada, 1 418 656-2131 ext 417857; 2 Department of Operations and Decision Systems, Faculty of Administrative Sciences, Laval University, Québec, QC, Canada; 3 iaelyon School of Management, Université Lyon 3, Lyon, France; 4 Department of Economics, University of Genova, Genova, Italy; 5 Marketing Department, Faculty of Administrative Sciences, Laval University, Québec, QC, Canada; 6 Library and Advisory Services Division, Laval University, Québec, QC, Canada

**Keywords:** Business Process Model and Notation, patient health care trajectories, implementation, effectiveness, transformation

## Abstract

**Background:**

Health care systems are increasingly confronted with the challenge of managing complex clinical processes. One proposed solution is a patient-centered management intervention called a care pathway that needs process mapping to support process improvement. Although the adoption and use of Business Process Modeling Notation (BPMN) for modeling patient health care trajectories has increased, evidence of the benefits of implementing it in health care organization management systems remains unclear.

**Objective:**

This review sought to examine effectiveness by mapping implementation factors linking intended purpose to expected or demonstrated outcomes.

**Methods:**

A systematic review of the use of BPMN for modeling patient health care trajectories was conducted across 8 databases. We followed the Cochrane Methods Group and the PRISMA (Preferred Reporting Items for Systematic Reviews and Meta-Analyses) guidelines. We selected empirical, experimental, and conceptual articles in French and English that analyzed and evaluated the implementation of BPMN for modeling patient health care trajectories in the health care field. Quality appraisal was performed using the Mixed Methods Appraisal Tool. Data were charted using a customized form and analyzed thematically with qualitative and semiquantitative syntheses.

**Results:**

After screening, 61 studies were included. BPMN offers significant benefits in health care. Its use allows health care professionals to gain an understanding of patient health care trajectories, making it easier to identify inefficiencies and areas for improvement. The definition of processes ensures that workflows remain consistent across different settings, thereby reducing variation and improving the quality of care. Several studies have demonstrated BPMN’s effectiveness in process optimization, highlighting its ability to streamline workflows, reduce redundancies, and enhance operational efficiency. Moreover, when integrated with decision-support tools, BPMN enhances clinical decision-making by enabling better adherence to guidelines and best practices. Another advantage is BPMN’s interoperability with existing health care IT standards. However, managerial considerations reveal trade-offs between BPMN’s benefits and limitations, especially in highly complex health care settings. Several challenges persist, including issues related to scalability, integration with advanced decision-making frameworks, and the complexity of modeling dynamic health care environments. While BPMN is widely adopted, alternative methodologies offer complementary or competing advantages. Several opportunities exist to enhance BPMN’s applicability in health care, such as creating domain-specific BPMN extensions or integrating artificial intelligence and machine learning. However, limitations in the methodological quality of the studies selected and the concentration of the research mainly in European countries limit the generalizability of our results.

**Conclusions:**

This review highlights BPMN’s potential as a valuable tool for modeling patient health care trajectories. Its ability to standardize and optimize processes makes it promising for improving clinical and operational efficiency. However, trade-offs between benefits and limits of BPMN characterize its implementation in patient health care trajectories, creating opportunities for the development and integration of new tools.

## Introduction

In many countries, health care systems are increasingly confronted with the challenge of managing complex clinical processes, where patient care often involves multiple departments, professionals, and stages [1]. In addition, health care systems are facing a rise in multimorbidity, along with a growing population of older adults. These conditions have significantly increased the public expectation for health care services, both in terms of volume and service quality [2]. In response, health care organizations have prioritized efficient and effective management practices to improve outcomes and outputs, all while managing resource and budget constraints [3]. Several national and international organizations have developed frameworks for improving the performance of health care and services on the dimensions of quality, safety, effectiveness, efficiency, patient-centeredness, timeliness, and equity [4-7]. These goals represent significant challenges that face any health care setting [8-10].

One proposed solution is a patient-centered management intervention called a care pathway [11-14], which is designed to guide patients, in a specific patient segment, through a trajectory of care representing the entire continuum of care, from prevention and screening to recovery or palliative care. These interventions are efficient in structuring and managing patient care, improving outcomes, accessibility, quality, sustainability, and cost-effectiveness [15-18]. However, the standardization, representation, and integration of the care pathway within management systems, including their ongoing process improvement, present further challenges for health care organizations.

During the last 2 decades, several tools have been developed to support process improvement through process mapping. Health care organizations began to adopt such tools as Business Process Modeling Notation (BPMN) to support various business processes, including the delivery of care [19,20]. Originally designed for business environments to represent processes as a network of activities and tasks [19], BPMN has proven to be an effective tool in health care by providing a standardized visual language for modeling processes [21] and has become one of the most important and widely used tools in this context [11]. Developed by the Object Management Group in 2004, BPMN has been adopted as an international standard by the International Organization for Standardization since 2012. Now in its second version, the BPMN 2.0 [20] enables the introduction of extensions that characterize specific domains (eg, health care, quality management, and security) consistent with original BPMN elements [22,23]. The main value of the extension is in the reuse of BPMN’s main functions, maintaining its standardization, without the need for developing domain-specific modeling languages [24].

Although the literature regarding the adoption and use of BPMN for modeling patient health care trajectories has increased over the last decade, existing reviews are limited. A preliminary search for existing reviews was conducted in the Cochrane Database, JBI Database of Systematic Reviews and Implementation Reports, and PROSPERO, and 4 literature reviews on BPMN in the health care context were identified [23,25-27]. The first, carried out in 2014, focused on clinical decision support [25]. The next 2 focused on the ability to formalize the process and standardized communication [26] or the possibility of incorporating variations or changes [27]. Finally, the last dealt only with extensions to the notation [23]. None of these reviews have addressed the effectiveness and characteristics of BPMN implementation nor the benefits and limitations of its use, particularly from a managerial perspective.

Thus, this study seeks to fill this gap and critically synthesize the empirical evidence on the uses of BPMN for modeling patient health care trajectories with a focus on implementation and effectiveness. We have adopted the Population, Intervention, Comparator, Outcomes, Timing, Settings (PICOTS) mnemonic criteria [28] in the development of our review objective and research questions. The objective of this systematic review is to examine the evidence linking the implementation of BPMN modeling of health care trajectories (I, P) to both management and clinical outcomes (O) in clinical health care settings (S). Within this objective, we seek to answer 2 specific research questions: how well do the objectives for using BPMN to model health care trajectories align with the realized outcomes, and what are the potential implementation factors (including the use of extensions) that link objectives to outcomes?

## Methods

### Overview

The protocol for this review was previously published as a scoping review protocol [29]. However, further development of the objectives and aims justified the conduct of a systematic rather than a scoping review. This systematic review was carried out in accordance with the Cochrane Methods Group [30] and the PRISMA (Preferred Reporting Items for Systematic Reviews and Meta-Analyses) guidelines [31]. Following them, we adapted the method from the previously published protocol by (1) reformulating the research question using PICOTS rather than Population Concept and Context, (2) excluding literature reviews from the article selection process, (3) incorporating a quality assessment, and (4) completing the PRISMA 2020 checklist for systematic reviews (Checklist 1).

### Information Sources and Search Strategy

The search strategy was developed in collaboration with an academic librarian specializing in medical and health care management fields (FB). Databases covering health (PubMed, Embase, and CINAHL) and business (ABI/INFORM) disciplines and multidisciplinary (Academic Search Premier, Web of Science, and ScienceDirect) databases, as well as the Google Scholar search engine, were searched to identify eligible peer-reviewed articles. The last search of each database was performed on January 5, 2026.

Key search terms were informed by previous relevant reviews and are shown in Table 1, mapped to the PICOTS framework. Searches in the electronic databases were conducted from January 1, 2004, as the BPMN was released in its first version to the public in May 2004. For Google Scholar, we limited results to the first 20 results per string and filtered out citations and patents. The full search strategy for each database is provided in Multimedia Appendix 1.

**Table 1.** Search terms mapped to PICOTS^a^ plus limits and filters.

PICOTS	Search terms
Patient population	Not specified
Intervention	Business Process* (Model* OR Method? OR management) OR Decision Model* notation OR BPMN* OR BPM
Comparator	Not specified
Outcomes	Not specified
Timing	Not specified
Settings	Critical Pathways OR Practice Guidelines OR Workflow OR Clinical Decision-Making OR Decision Support Systems, Clinical OR Patient Care Management
Settings	Decision (making OR support) OR (clinical OR medical OR healthcare OR “health care”) process* OR (healthcare OR clinical OR critical OR care) path* OR guideline* OR Workflow* OR careflow* OR patient journey OR Healthcare trajectory
Other limits	Dates: January 1, 2004 to present (last search January 5, 2026) For ABI/INFORM (ProQuest): peer-reviewed publications only For Google Scholar: first 20 results per string only; filtered out citations and patents

a PICOTS: Population, Intervention, Comparator, Outcomes, Timing, Settings.

### Eligibility Criteria

The eligibility criteria aligned with the PICOTS are provided in Table 2.

**Table 2.** Eligibility criteria mapped to the PICOTS^a^ framework.

PICOTS	Inclusion criteria	Exclusion criteria
Patient population	No limits within patient health care trajectories	—^b^
Intervention	Related to BPMN^c^ in health care trajectories Models a care process ideally citing BPMN	Not related to BPMN in health care Clearly specifies the exclusive use of a notation other than BPMN (such as UML^d^, BPEL^e^, HL7^f^, ARIS, etc) Addresses another type of business process management other than process modeling Computer programming and coding (eg, focus on HL7 or other standards)
Comparator	No limits	—
Outcomes	Any indicator to estimate the improvement in process management or clinical outcome	—
Timing	No limits	—
Settings	Explicitly in the health care field Health care trajectories or clinical or care pathways	Theoretical demonstration of a new tool Evaluation process of a tool without application (eg, metrics)
Other limits	Articles in French and English only	Theses and dissertations Editorials Literature reviews and protocols

a PICOTS: Population, Intervention, Comparator, Outcomes, Timing, Settings.

b Not applicable.

c BPMN: Business Process Modeling Notation.

d UML: Unified Modeling Language.

e BPEL: Business Process Execution Language.

f HL7: Health Level Seven.

### Study Selection

The search results were imported to Covidence (Veritas Health Innovation Ltd) [32] to remove duplicates and manage the study selection process. Study selection proceeded in 2 phases, beginning with a title and abstract screening, followed by a full-text review of retained records. At each phase, a pair of reviewers consisting of 2 of the 3 coauthors (JBG, PL, and MTH) independently screened the titles and abstracts (phase 1) or reviewed full texts (phase 2) against the inclusion and exclusion criteria. Conflicts were managed by discussion to reach a consensus, or, when necessary, an additional reviewer (AC) was consulted.

### Data Extraction and Data Items

A pair of reviewers consisting of 2 of the 4 coauthors (PL, JBG, MTH, and CL) independently extracted data from the included studies using a custom-developed data extraction form in Microsoft Excel which included the following data fields: “citation details” (title, authors, year of publication, and author affiliations), “study description” (study design, setting, care trajectory, aims or objectives, key variables analyzed, and extensions to BPMN used), “study results” (findings, outcomes, and study limitations), “BPMN utility” (objective for use, benefit or advantage of use, limit of use, opportunities, and alternatives or threats). JBG and PL reviewed all the data extraction tables, presented in Multimedia Appendix 2. Discrepancies were addressed through discussion between at least 2 reviewers.

### Quality Assessment

Quality assessment was performed using the Mixed Methods Appraisal Tool (MMAT) of July 2020 [33], which uses five core quality criteria to assess each of the following study designs: (1) qualitative, (2) randomized controlled, (3) nonrandomized, (4) quantitative descriptive, and (5) mixed methods. Assessments were performed independently by 2 reviewers (MTH and JBG). Disagreements were addressed through discussion and, where needed, a third independent assessor (PL or AC) was consulted. A 3-level summary of quality was assigned to each study based on the number of achieved criteria, where 5=high quality, 3‐4=medium quality, and ≤2=low quality. For mixed methods studies, the lowest scoring of 3 study design sets (qualitative, quantitative, or mixed) was used to assign the summary level. Due to contention in the literature about the use of summative approaches in critical appraisal [34-37], we also provide a detailed presentation of the ratings (refer to Table 3 in the “Results” section). Studies that failed the MMAT screening questions S1 or S2 due to a lack of a clear research question were categorized as “Experience feedback” and were not formally appraised for quality.

**Table 3.** Results of the quality assessment with the MMAT^a^ tool.

Title	Experience feedback (no research question)	Qualitative studies	Quantitative studies	Mixed methods studies	MMAT quality level
Number of studies	46	4	3	8	
Distribution of quality scores					
0	—^b^	—	—	2	Low quality
1	—	—	—	—	Low quality
2	—	—	1	—	Low quality
3	—	—	1	1	Medium quality
4	—	1	—	4	Medium quality
5	—	3	1	1	High quality

a MMAT: Mixed Methods Appraisal Tool.

b Not applicable.

### Data Analysis and Synthesis

Descriptive data items were reduced to meaningful categories as presented in Table 4. The characteristics of the included studies were analyzed descriptively, and we used multidimensional scaling in Orange (v3.38; Bioinformatics Lab) to visualize the relations within the body of literature across 3 dimensions: geography, study design, and study (health care) setting.

The text data extracted in the “Study Results” and “BPMN utility” fields underwent inductive thematic coding. This was done by examining extracts for keywords and phrases, grouping like keywords and phrases into subthemes, and further grouping those subthemes into broader themes to generate a 2-level coding tree by which extracts were coded.

**Table 4.** Characteristics of the selected studies.

Characteristic and category	Value, n (%)	References
Publication year		
2008/09	3 (4.9)	[38-40]
2010/11	0 (0.0)	—^a^
2012/13	3 (4.9)	[41-43]
2014/15	11 (18.0)	[44-54]
2016/17	13 (21.3)	[55-67]
2018/19	9 (14.8)	[68-76]
2020/21	6 (9.8)	[77-82]
2022/23	8 (13.1)	[83-90]
2024/25	8 (13.1)	[91-98]
Geography		
Africa	4 (6.6)	[56,62,63,84]
Asia	3 (4.9)	[55,64,80]
Europe	42 (68.9)	[38-49,51-54,57,58,60,61,65,66,68,69,71-74,77,79,82,83,85-90,92-95,97]
Middle East	3 (4.9)	[67,91,98]
North America	3 (4.9)	[47,59,76]
South and Central America	6 (9.8)	[50,70,74,78,83,96]
Study design		
Empirical	30 (49.2)	[43,51-53,64,67,70,72,74,75,79-98]
Experimental	20 (32.8)	[41,42,44-47,54,56,57,60,62,63,65,66,69,71,73,76-78]
Conceptual or theoretical	11 (18.0)	[38-40,48-50,55,58,59,61,68]
Study setting		
Hospital	29 (47.5)	[41-43,45,48,52,54-56,58,64-72,74,76,79,83,84,86-88,91,94]
Integrated care	11 (18.0)	[38,40,53,60,73,75,78,81,92,96,97]
Specialist or multidisciplinary outpatient clinic	6 (9.8)	[47,80,82,85,93,95]
Primary care	5 (8.2)	[49,63,77,89,90]
Emergency department	5 (8.2)	[39,44,51,57,62]
Home care	1 (1.6)	[98]
Undefined	4 (6.6)	[46,50,59,61]
Care trajectory		
Acute care	27 (44.3)	[39,41,44-46,51,54,57-59,62,64,66,67,71,74,78,82-87,91,94,95,97]
Chronic care	20 (32.8)	[40,43,47-49,52,56,60,63,65,68,70,75,77,79,81,88-90,93]
Integrated care	6 (9.8)	[42,53,73,80,92,96]
Preventive care	2 (3.3)	[69,76]
Other	6 (9.8)	[38,50,55,61,72,98]
BPMN^b^ extension use		
Yes	48 (78.7)	[38-60,62,63,65,66,68-73,75-78,80,82,84-89,92,95,96]
No	11 (18.0)	[67,74,79,81,83,90,91,93,94,97,98]
Unclear	2 (3.3)	[61,64]
BPMN+ extension and additional tools or standards		
ABC^c^	2 (3.3)	[73,77]
BPM^d^ lifecycle	1 (1.6)	[62]
BPMN4CP^e^	6 (9.8)	[40,46,50,51,56,58]
BPMN+V^f^	2 (3.3)	[75,88]
BPSim^g^	2 (3.3)	[57,95]
CPG^h^	5 (8.2)	[38,48,52,63,69]
Clinical safety checklist	1 (1.6)	[64]
DMN^i^	5 (8.2)	[45,60,68,76,87]
EVALAB graphical interface	1 (1.6)	[44]
FHIR^j^	3 (4.9)	[85,89,96]
HACCP^k^	1 (1.6)	[72]
ICT^l^	6 (9.8)	[39,41,47,53,55,59]
IT sensor	1 (1.6)	[65]
PROforma SIG	1 (1.6)	[90]
UML^m^	1 (1.6)	[54]
VSM^n^	2 (3.3)	[43,84]
BPMN ontology and SWRL^o^	1 (1.6)	[49]
BPMNsix^p^+ IEEE 11073 SDC	1 (1.6)	[71]
ICNP^q^+ computational tools (Bizagi Modeler Software)	1	[78]
t.BPMN^r^+ BPMN4 CP	1	[42]
DMN + CMMN^s^	3	[66,80,86]
DMN+ FHIR	1	[82]
eHealth and ubiquitous computing and knowledge management and clinical decision-support systems	1	[70]
HL7+^t^ FHIR	1	[92]
Unknown	1	[61]
None	10	[67,74,79,81,83,91,93,94,97,98]

a Not applicable.

b BPMN: Business Process Modeling Notation.

c ABC: Activity-Based Costing.

d BPM: Business Process Management.

e BPMN4CP: BPMN for clinical pathways.

f BPMN+V: a data-enriched subset of BPMN1 suitable for modeling clinical guideline.

g BPSim: Business Processes Simulation 1.0.

h CPG: clinical practice guideline.

i DMN: Decision Modeling Notation.

j FHIR: Fast Healthcare Interoperability Resource.

k HACCP: Hazard Analysis and Critical Control Point.

l ICT: Information and Communication Technology.

m UML: Unified Modeling Language.

n VSM: Value Stream Map.

o SWRL: Semantic Web Rule Language.

p BPMNsix + IEEE 11073 SDC: BPMN + Surgical Intervention Extension.

q ICNP: International Classification for Nursing Practice.

r t.BPMN: tangible Business Process Modeling.

s CMMN: Case Management Model Notation.

t HL7: Health Level Seven.

Frequency analyses of both broad and subthemes were performed on the BPMN benefits, limits, opportunities, and alternatives or threats variables to determine the most prevalent themes represented in the literature in these 4 domains. We then constructed 2 composite variables of thematic frequencies, one representing limits and benefits as a continuum (benefit-limit), and the other, opportunity and threat as a continuum (opp-threat). To do this, we negatively transformed the limit and threat frequencies to represent the negative sides of the continuums, whereas benefit and opportunity were represented as positive values. We then plotted opp-threat (*y*-axis) against benefit-limit (*x*-axis) to examine how limits and benefits related to threats or opportunities at a broad thematic level. We then narratively examined the subthemes in relation to the predominant relationships between broad themes in the plot and synthesized this with a narrative analysis of the major themes from the study findings fields.

Finally, we mapped this synthesis to a proposed causal pathway describing the mechanisms by which the purpose or objective for using BPMN to model patient health care trajectories links to desired (or observed) outcomes through characteristics of BPMN implementation expressed as benefits, limits, opportunities, and threats or alternatives. The mechanisms have been classified according to the domains of the Consolidated Framework for Implementation Research (CFIR) [99-101].

## Results

### Overview

**Figure 1.**
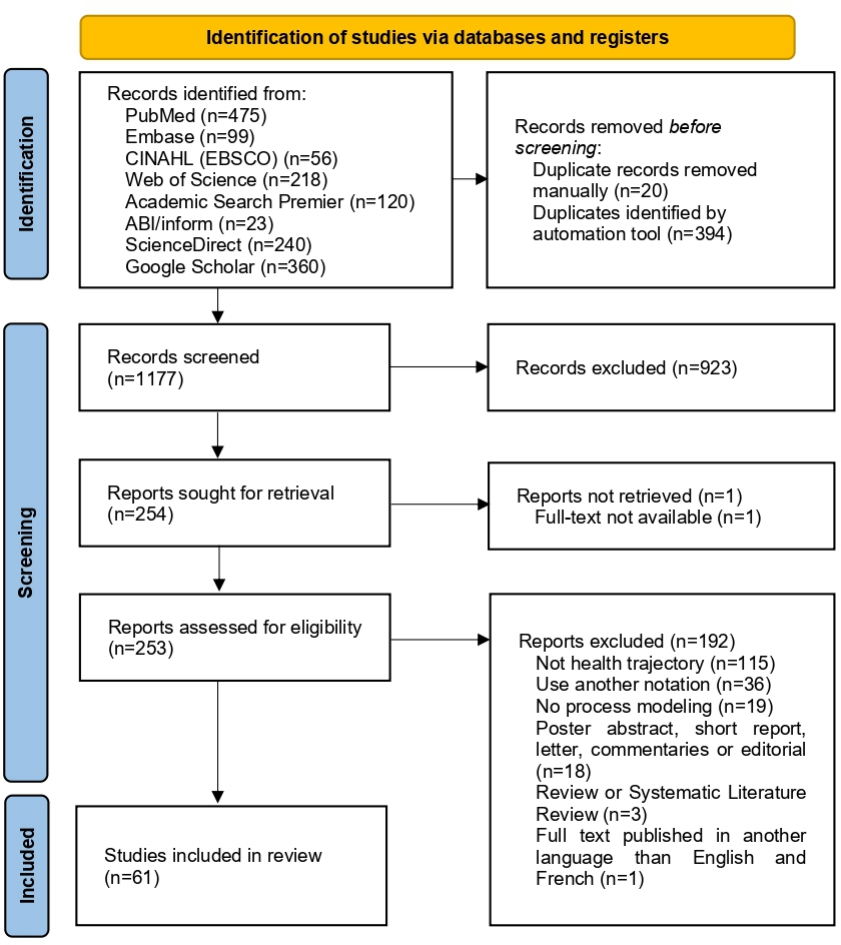
PRISMA (Preferred Reporting Items for Systematic Reviews and Meta-Analyses) 2020 flow diagram of the systematic review process.

Following the identification and removal of duplicates, 1177 unique records were identified. Screening against the eligibility criteria resulted in the retention of 253 studies, of which a further 192 were excluded by full-text review, resulting in 61 included studies [38-98]. This process is shown in the PRISMA 2020 flow diagram in Figure 1.

### Study Characteristics

The characteristics of the selected literature are summarized in Table 4. The first published study of BPMN in modeling patient health care trajectories was in 2008 [38]. Most studies were performed in the European context and were conducted in a hospital setting, addressing a range of health care trajectories across acute and chronic care. The concentration of studies in Europe may be considered a bias for the validity of the study results.

**Figure 2.**
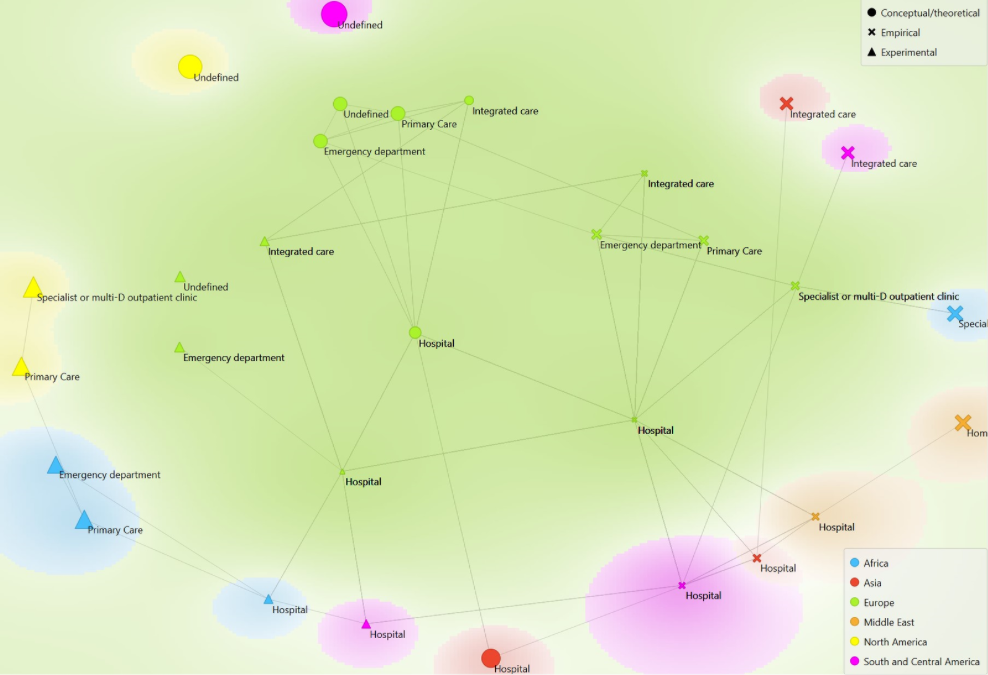
A relational map of the selected literature in terms of geography, study design, and study (health care) setting.

A relational map of the selected literature in terms of geography, study design, and study (health care) setting is shown in Figure 2. These three dimensions are illustrated in the figure as follows: geography (marker colors), study design (marker symbols), and study (health care) setting (marker labels). The more tightly clustered a group of papers is, the more closely they are related to each other across these 3 dimensions. The size of the marker symbols is scaled to the goodness-of-fit of each case, where the smaller the marker symbol, the better the fit. This analysis shows the clear dominance of Europe (indicated in green) in the development of the evidence base from conceptual or theoretical roots (circle markers) through empirical (x markers), including experimental investigation (triangle markers). It is noteworthy that at each stage of this literature development, evidence was contributed from a range of health care settings (hospital, emergency departments, primary care, and integrated care), reducing the risk of limitations to knowledge development arising from setting specificity. In contrast to this European dominance in the development of the literature, this analysis also suggests that the rest of the world has proceeded with empirical and experimental research based on European experience, with only a few examples of conceptual or theoretical research having been conducted outside of Europe. Like within Europe, the research outside of Europe represents a diverse evidence base developed from hospital, primary care, integrated care, emergency department, and specialist or multidisciplinary health care clinic settings.

### Quality Assessment

Table 3 presents the quality assessment using the MMAT tool of July 2020 [33]. The full scoring can be seen in Multimedia Appendix 3. Overall, the number of methodologically high-quality studies is low, which limits the scope and generalizability of our study’s results.

### Objectives for Using BPMN

The primary objective of using BPMN was to formalize care processes by modeling and visualizing them. Indeed, BPMN modeling of the patient health care trajectory involved the visual representation of workflow models and activities [41,42,44,46-49,51,54,62,64,66-70,77,80,83,85], roles and systems within care pathways [45,50,55,57,70,93,98]. Further, such representation was used to validate the workflow with the participants in the field [63] and even to outsource certain processes [40].

Some studies aimed to integrate dedicated extensions in predefined dimensions to overcome shortcomings of BPMN. These extensions had different objectives, such as (1) integrating decision support via Decision Modeling Notation (DMN) rating [72,85], or in combination with Case Management Model Notation (CMMN) [69], both developed by Object Management Group; (2) modeling clinical practice guidelines (CPGs) [80]; (3) incorporating the notion of value through the Value Stream Map notation derived from lean management [46,87]; (4) knowledge definition for a rich and expressive graphical representation [59]; (5) the Hazard Analysis and Critical Control Points [75]; (6) a dynamic approach to BPMN [82]; or (7) adaptation to specific process contexts such as modeling clinical pathways [55,61,93,95,97], or operating room processes [74].

Beyond simple process formalization, patient health care trajectory modeling using BPMN is also used to analyze existing processes, visualize constraints, and simulate changes to optimize operational performance. A comprehensive analysis [70] of existing processes using BPMN shows that it enables a specific focus to be placed on the resources and activities, to incorporate them as constraints of the optimization model [45] and to define opportunities for redesigning the process [53,60,65,70,74]. More recently, BPMN has been used to provide a set of recommendations for clinical or care pathway optimization [46,87,91,92,94,95,97,98], sometimes in support of value-based health care [98]. In addition, BPMN modeling can then be used to simulate different scenarios for improvement [57,61,91,95] and provide proof of concept for possible optimization results [59]. However, using BPMN does not necessarily enable the initial objectives to be achieved and can lead to a variety of benefits, limitations in its use becoming opportunities for optimization or even giving rise to alternatives that could become threats.

### Benefits, Limits, Opportunities, and Alternatives or Threats

A total of 26 broad themes and 59 subthemes were identified in the BPMN utility data fields. Table 5 shows the distribution of extractions across each of these broad themes for each domain of benefits, limits, opportunities, and alternatives or threats. The italic text indicates the most prominent themes, using 10% representation of column dimensions (benefits, limits, opportunities, and alternatives or threats) as an arbitrary cutoff. These are used to map the BPMN Purpose-Implementation-Outcome Model in the next section.

**Table 5.** Themes related to expressed benefits, limitations, and opportunities associated with the use of BPMN^a^ in health care, as well as identified alternatives or threats to its use.

Theme	Benefits	Limits	Opportunities	Alternatives or threats
Accessibility	2 (0.9)	0.0	0.0	0.0
*Automation and conditionality*	2 (0.9)	2 (2.0)	8 (4.8)	*8 (14.8)* ^b^
*Clinical utility*	19 (8.6)	9 (9.2)	*20 (11.9)*	0.0
Collaboration	11 (5.0)	1 (1.0)	5 (3.0)	0.0
*Comprehensiveness*	18 (8.1)	*18 (18.4)*	7 (4.2)	2 (3.7)
Customization	0.0	2 (2.0)	0.0	0.0
*Data and measurement capability*	10 (4.5)	*13 (13.3)*	*21 (12.5)*	2 (3.7)
*Decision-making*	2 (0.9)	1 (1.0)	14 (8.3)	*9 (16.7)*
Efficiency	1 (0.5)	5 (5.1)	1 (0.6)	1 (1.9)
Extensibility	4 (1.8)	2 (2.0)	4 (2.4)	0.0
Flexibility	9 (4.1)	0.0	2 (1.2)	1 (1.9)
*Health care suitability*	6 (2.7)	9 (9.2)	4 (2.4)	1 (1.9)
Information management	0.0	2 (2.0)	7 (4.2)	5 (9.3)
Integration	2 (0.9)	1 (1.0)	4 (2.4)	0.0
Interoperability	2 (0.9)	1 (1.0)	3 (1.8)	1 (1.9)
*Language utility*	*49 (22.2)*	7 (7.1)	6 (3.6)	5 (9.3)
Machine interpretability	7 (3.2)	0.0	1 (0.6)	0.0
*Management utility*	*27 (12.2)*	*10 (10.2)*	11 (6.5)	0.0
Optimization	1 (0.5)	0.0	4 (2.4)	0.0
Process utility	5 (2.3)	3 (3.1)	6 (3.6)	3 (5.6)
Scalability	0.0	0.0	0.0	3 (5.6)
*Simulation*	4 (1.8)	2 (2.0)	5 (3.0)	*6 (11.1)*
Supportive technology	0.0	2 (2.0)	6 (3.6)	0.0
*Tools*	1 (0.5)	0.0	*19 (11.3)*	3 (5.6)
User experience	19 (8.6)	8 (8.2)	3 (1.8)	2 (3.7)
Visualization	20 (9.0)	0.0	1 (0.6)	2 (3.7)
Total articles	61	61	61	61
Articles without relevant extractions	7	13	10	42
Total extractions (denominator)	221	98	168	54
Mean extractions per paper	3.6	1.6	2.8	0.9
Data =extraction counts (column %)				

a BPMN: Business Process Model and Notation.

b The italicized text indicates the most prominent themes, using 10% representation of column dimensions (benefits, limits, opportunities, alternatives or threats) as an arbitrary cut-off. These are used to map the BPMN Purpose-Implementation-Outcome Model in the next section.

**Figure 3.**
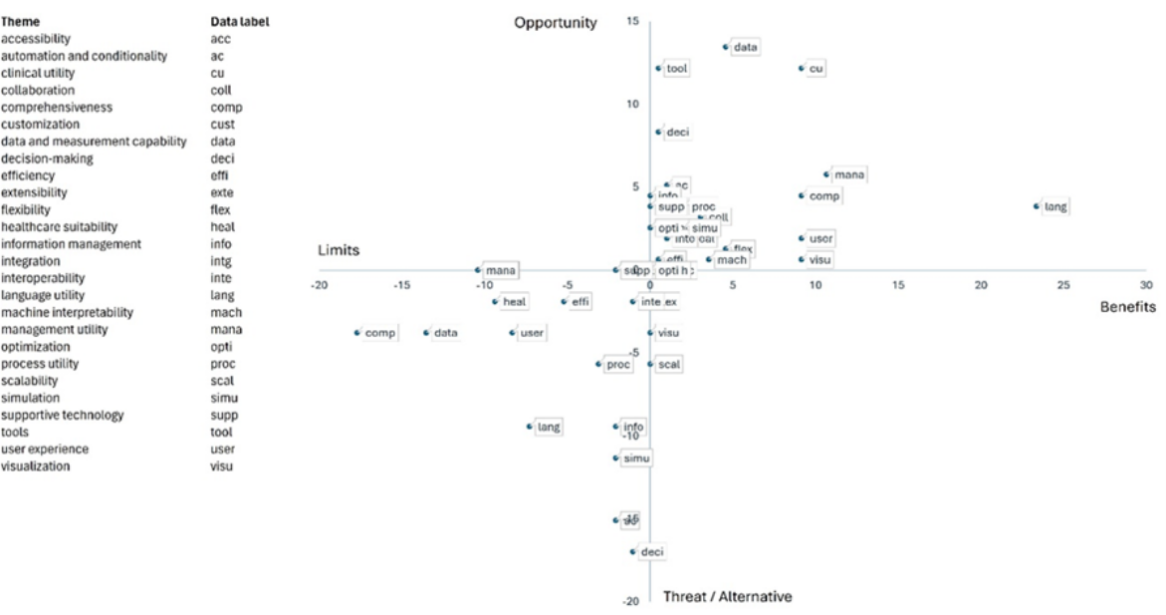
Relational frequencies of themes plotted by benefit-limitation against opportunity-threat.

These frequencies were then used to plot opportunity-threat against benefit-limit to illustrate where benefits or limits might relate to opportunities or threats across all themes in a relational frequencies of themes plotted by benefit-limit against opp-threat figure (Figure 3). For this analysis, benefits (positive) and limitations (negative) were considered as representing positions along the same continuum (*x*-axis) as were opportunities (positive) and alternatives or threats (negative; *y*-axis). Data represent column percentages presented in Table 3 using negative transformed values for both limitations and alternatives or threats. Positions right of the vertical represent benefits, left of the vertical represent limitations. Positions above the horizontal represent opportunities and below the horizontal represent alternatives or threats.

To unpack the relationships between benefit-limit and opportunity-threat at the broad theme level, that is, to better understand, for example, where limits represent opportunity and where they represent threat for BPMN, we analyzed the distribution of subthemes within the dominant broad themes (Multimedia Appendix 4).

### A BPMN Purpose-Implementation-Outcome Model

#### Overview

This narrative synthesis is structured as a model that seeks to describe how BPMN use case (purpose or objective) links to outcomes through characteristics of BPMN implementation expressed as benefits, limits, opportunities, and threats or alternatives.

First, to revisit the primary objectives for using BPMN to model patient health care trajectories, we saw that formalization of care processes by modeling and visualizing the processes within patient health care trajectories was the dominant objective, followed by analyzing existing processes, visualizing constraints, and simulating changes to optimize operational performance. These objectives sought to create improved clinical outcomes at both the level of the individual patient and for the organization’s performance. Indeed, some projects have demonstrated impacts on the organization of health care services for improved quality and safety. These outcomes translated into an improvement in patient outcomes [41,44,47,61,64,76,78,81,82,84], such as treatment success [41,61,81,82], and organizational performance outcomes such as service quality [61,82], expressed in the literature as reduced delays in diagnosis and treatment [44], reduction in medical errors [47,78], reduced unnecessary appointments and duplicated processes [84], reduced redundancies in clinician workflows [47,64,76], and improving patient autonomy in decision-making [81].

**Figure 4.**
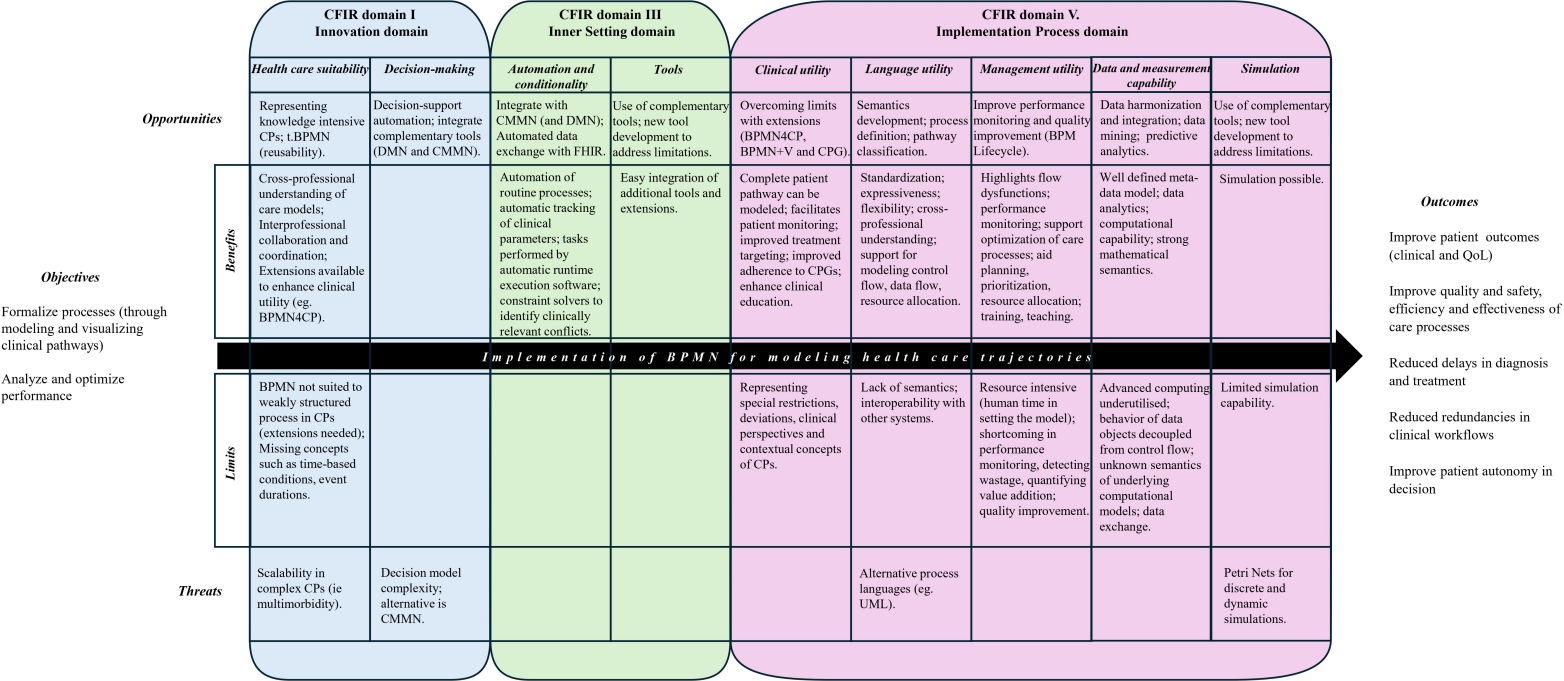
A Business Process Model and Notation purpose-implementation-outcome model. BPMN: Business Process Model and Notation; BPMN+V: a data-enriched subset of BPMN1 suitable for modeling clinical guidelines; BPMN4CP: BPMN for clinical pathways; CMMN: Case Management Model Notation; CP: clinical pathway; CPG: clinical practice guideline; CFIR: Consolidated Framework for Implementation Research; DMN: Decision Modeling Notation; FHIR: Fast Healthcare Interoperability Resource; QoL: Quality of Life; t.BPMN: Tangible Business Process Modeling; UML: Unified Modeling Language.

We now move to unpack the implementation mechanisms by which BPMN, used for the above-stated objectives, may facilitate or inhibit the realization of these anticipated and demonstrated outcomes. To do so, we adopt a logic model to map out these mechanisms within the dominant broad themes outlined in Table 5 and Figure 4. The thematic analyses were synthesized in accordance with the CFIR domains [99-101].

#### CFIR Domain I: Innovation Domain

The CFIR domain I corresponds to the key characteristics of the innovation implemented regardless of the implementation process or adaptation carried out [100].

##### Health Care Suitability

The literature highlights both benefits and limitations of BPMN in health care but presents minimal discussion on opportunities and threats to its suitability. BPMN facilitates understanding of care models among professionals [42,44,55,65,76,83-85,87,90-92], formalizes organizational knowledge [40], and complex health care processes [40,59], and enables in-depth process analysis and cross-professional understanding of care models by clinicians [73,90-92], IT staff [40,45,50,54,60,64,65,71,74,75,78,87], and process experts [53,54,60,65,74].

It enhances interprofessional collaboration between care professionals [49,78,90,98] and with support services such as IT [59] by involving various stakeholders in coordination. Indeed, involving the various stakeholders in care process modeling improves coordination in care processes by describing collaboration modes [40,49] and communication processes [41,44,98].

While BPMN supports complex health care trajectories [64] and can be extended for enhanced functionality [71], its core limitations necessitate extensions [54,61], particularly for time-based conditions and event durations [84,87]. Aissaoui and colleagues [84] highlight its ability to model diverse patient pathways, whereas Wiemuth and colleagues [66] note its limitations in capturing weakly structured processes. Scalability remains a challenge, especially in modeling the highly complex care pathways navigated by patients with multimorbidity [59]. However, opportunities exist for developments in representing knowledge-intensive clinical pathways [61]. For instance, modular construction systems such as t.BPMN (tangible Business Process Modeling) offer potential for enhanced interprofessional analysis [42], and model reusability or replication, which have been noted as limitations [63,69,82,90].

##### Decision-Making

Without extensions, BPMN cannot model decisions and provide guidance for their application and rules [64,68,76]. However, BPMN offers opportunities in automated decision support [52,96] and integration of DMN and CMMN for decision execution [60,66,68,86] in different health care contexts, including surgery [71]. CMMN is particularly suited for unstructured processes requiring real-time adaptability [58,98]. However, conclusive results are still lacking.

BPMN’s limitations in modeling complex decision scenarios [61] create demand for alternatives such as CMMN, which enables dynamic, condition-based task activation [66].

### CFIR Domain III: Inner Setting Domain

The CFIR domain III corresponds to the structural characteristics of the setting, including the existing IT infrastructure [100].

#### Automation and Conditionality

BPMN facilitates automation, task tracking, and interoperability via continuous communication based on Health Level Seven (HL7) integration [41,72,98], a set of international standards designed to facilitate the exchange, integration, sharing, and retrieval of electronic health information between disparate medical apps. It supports capacity-based task allocation [59,78], integration of runtime execution software [64,66,74,75], and automated patient monitoring [81]. While BPMN struggles with repeatable task modeling [66], integration with CMMN and DMN enhances automated and conditional advanced decision-making and task execution [66].

Further opportunities include automatic data exchange with hospital information systems and electronic health records (EHRs) via Fast Healthcare Interoperability Resources (FHIR) [81,92,96], a modern, web-based standard from HL7 International for electronically exchanging health care data.

#### Tools

BPMN integrates well with complementary tools such as DMN [61,66,68,87] and CMMN [79] to enhance decision modeling, and FHIR [89,92,96] to interact with clinical data systems and EHRs. Additional tools facilitate checklist implementation [64] and surgical decision processes [71]. Calls for artificial intelligence (AI)–driven process apps [55,97] and advanced software for real-time execution [55,89] present further opportunities for BPMN tool development in health care.

### CFIR Domain V: Implementation Process Domain

The CFIR domain V corresponds to the activities and strategies used to implement the innovation and their impacts on practices [100].

#### Clinical Utility

BPMN contributes to clinical effectiveness [81], clinical guideline adherence [52,82], patient monitoring [81,96], clinical education [79,81], clinical safety [78], and quality care [82]. It aids in translating CPGs into logical models [87,88,90,91], integrating recommendations at every step of the care trajectory [48], and supporting clinical decision-making [47,52,60,68-70,86]. BPMN allows clear process flows with treatment steps, often condition-based [84], to be described precisely [87], though integration of an extension capable of capturing the sophistication of CPGs [88] may be superior.

Without extensions, BPMN cannot (1) model decisions, (2) provide guidance for their application and rules [64,68,76], (3) account for variations in procedures or trajectory deviations to include multiple perspectives [46,59,60,66,81], (4) integrate specific knowledge such as guidelines or contextual knowledge such as specific roles for resources, activities [52,56,71,81], or responsibilities [81]. In addition, it is difficult to model roles for shared activities [58], value addition [84], or specific delays and time constraints [54,61,66,84,87]. DMN and CMMN together address some of these challenges, enabling decision representation and structured workflow execution [64,68,76]. Advances such as BPMN extension for clinical pathways [46,59] and tools such as BPMN+V (a data-enriched subset of BPMN1 suitable for modeling clinical guidelines) improve precision in process definition and patient-specific pathway navigation [88].

#### Language Utility

As an industry-standard process modeling language, BPMN integrates tasks, events, and gateways [71], but lacks formal semantics, complicating system interoperability [75,81,89]. This limitation impacts data interaction with the control flow [75,88] and the content of rules [64]. Unified Modeling Language is frequently cited as an alternative [53,72,77], but BPMN has the potential to be enhanced through semantics development [39,48], specifically to develop operational semantics based on partially ordered events to allow the integrated execution of multiple process models at the same time to recommend treatment steps [48].

#### Management Utility

BPMN supports health care management [47,50,54,73,78,82,83,85,91-98] by improving process understanding [47,54,83,85,91-95,97] and performance monitoring [57,67,78,80,81,94,96,97]. When combined with DMN, it can also improve compliance with clinical guidelines and best practices [52,64,68,76,82], and therefore improve quality of care as measured by treatment outcomes, cost reduction, resource planning, exception management, and coordination between organizational units [82]. Tomaskova and colleagues [73] emphasized BPMN’s benefit to the management functions of public administrations through its ability to distinguish social from health care processes and outcomes, including by disease stage.

BPMN highlights inefficiencies, including inadequate delays [43,44,61,91-95,97], process criticalities [57], redundant activities [44,48,81], and documentation [44,48]. BPMN-based dashboards track service quality [45,55,74,80], patient waiting lists [45], and resource use [45,50,61], such as operating room efficiency [45]. However, BPMN lacks native support for process measurement and value quantification [84]. Extensions such as Conformance Checking [72] improve quality assessment, audit processes, and predictive analysis, while BPM lifecycle enhancements refine process model evaluation [62].

#### Data and Measurement Capability

BPMN faces challenges in computational models [39], data exchange [82], and advanced computing [78,81]. Underuse of technologies such as AI and predictive analytics restricts their measurement potential [81]. Rodrigues et al [78] attribute this, in part, to limited IT exposure among nursing professionals.

Despite this, BPMN enables integration of data objects (documents) [58], computational simulations [42,44], and structured metadata [56]. Opportunities exist in harmonizing data structures [52,76,82], AI-driven patient trajectory prediction [79], and linking BPMN with health economics evaluation [73]. Tomaskova and colleagues [73] highlight its potential in assessing new cost-effective treatments, while Bianchi and colleagues [82] advocate for a shared data model across BPMN, DMN, and other standards to facilitate this at scale. More recently, some authors have highlighted the potential of BPMN for measuring efficiency and capacity [94,96], risk [94], and tracking and comparing patient outcomes through Key Performance Indicators [96].

#### Simulation

An interesting aspect of the use of BPMN is its ability to allow the simulation of different scenarios. Simulation helps define the solution best suited to the situation and context [57,72,91,95], highlighting the diversity of issues and constraints [43,44,61] and clarifying potential improvement and impact of performance [42,57,73,91,95].

However, simulation appeared to be considered almost exclusively as a threat to BPMN in the early published articles [38,39], with Petri Nets being considered a major alternative for discrete and dynamic simulations [38,39] and an important candidate for designing and implementing clinical services [38].

## Discussion

### Principal Findings

This systematic review provides an in-depth examination of the effectiveness of BPMN in modeling patient health care trajectories. Our findings suggest that BPMN has a more pronounced contribution to a managerial approach than to clinical relevance. Indeed, the use of BPMN improves process comprehension [47,53,83,86,91-98] while also creating opportunities for optimizing patient outcomes [41,61,76,81,92,94,96] and organizational performance outcomes [43,47,61,64,76,78,82,84,91-98]. However, despite these advantages, several challenges persist, including issues related to scalability [59], integration with advanced decision-making frameworks, and the complexity of modeling dynamic health care environments [61,66] or less structured processes [61,66,98].

### Benefits of BPMN in Health are

One of the principal benefits of BPMN in health care is its ability to facilitate workflow visualization [41,42,44,46-49,51,54,62,64,66-70,77,80,83,85,92,93,98]. This capability allows health care professionals to gain a comprehensive understanding of patient health care trajectories [42,44,55,65,76,83-85,87,90-93], making it easier to identify inefficiencies [43,44,48,57,61,81,91-95,97] and areas for improvement [53,60,65,70,74,91-98]. The definition of processes ensures that workflows remain consistent across different settings [84,87], thereby reducing variation and improving the quality of care [52,78,79,81,82]. Several studies have demonstrated BPMN’s effectiveness in process optimization, highlighting its ability to streamline workflows [43], reduce redundancies [47,64,76], and enhance operational efficiency [45,50,61,94]. Moreover, when integrated with decision-support tools such as DMN, BPMN enhances clinical decision-making by enabling better adherence to guidelines and best practices [52,64,68,76,81,91]. Another important advantage is BPMN’s interoperability with existing health care IT standards, such as HL7 [41,72,92] and FHIR [81,89,92,96], which facilitates seamless integration with EHRs and other digital health systems [64,66,74,75,96]. However, it is interesting to note the lack of consideration given to the “social dimension” of transforming practices associated with any transformation project, such as those using BPMN notation, despite this dimension being widely recognized in implementation frameworks such as the CFIR that we have used.

### Challenges

Despite its many advantages, BPMN also has notable limitations that impact its applicability in health care settings. One of the most significant challenges is its complexity, particularly when modeling highly dynamic, multistakeholder environments, such as long-term chronic disease management [61,66]. The ability of BPMN to represent decision-making processes is limited [64,68,76], requiring extensions such as DMN and CMMN to capture complex, evolving patient pathways [60,66,68,87]. Another critical challenge is BPMN’s limited capability to integrate real-time data analytics and AI for predictive modeling, an area of growing importance in modern health care [58,78,81,97]. Additionally, BPMN requires a specialized skill set [78], meaning that health care professionals need dedicated training, a potential barrier to widespread adoption.

### Opportunities for Future Development

Despite these limitations, there are several opportunities for enhancing BPMN’s applicability in health care. One key area is the creation of domain-specific BPMN extensions [27], which can improve its ability to represent complex clinical pathways. Another promising avenue is the integration of AI and machine learning into BPMN models, allowing for more sophisticated predictive analytics and decision automation [78,81,97]. Enhancing interoperability by developing standardized data exchange formats between BPMN and health care IT systems could further improve system efficiency and adoption [98]. Additionally, BPMN could benefit from the augmentation of dynamic simulation tools [42-44,57,61,72], which would allow for a more accurate representation of complex patient trajectories. This is why, from a managerial perspective, the use and implementation of the BPMN tool have definite potential, but its implementation and results remain uncertain. Indeed, the implementation of this technical tool suffers from a lack of support for a social approach to transforming practices and understanding the complexity of such projects, which Madan [97] describes as the perceived lack of consideration for the human dimension in the transformation of processes. That is why we recommend considering the social dimension necessary for any organizational change by integrating the “individual domain,” the IV domain that exists in the CFIR [100] and which is absent from the studies analyzed. From this perspective, a recent framework for implementing care pathways proposes integrating BPMN as a reflective tool to support the optimization and transformation of organizational and clinical practices, rather than as a tool for standardizing clinical processes [14].

### Alternatives and Competing Approaches

While BPMN is a widely adopted modeling approach, alternative methodologies offer complementary or competing advantages. Petri Nets, for instance, provide a powerful framework for discrete-event simulation and dynamic process modeling, which may be more suitable for certain applications [38,39]. Unified Modeling Language offers a robust structural representation [53,72,77,98] but lacks BPMN’s process-focused approach [39,48]. Similarly, Business Process Execution Language is more suited for automated workflows but does not provide BPMN’s visual representation capabilities [46]. A hybrid approach, integrating BPMN with AI-driven decision support systems and big data analytics, could help bridge some of these gaps and enhance BPMN’s clinical applicability.

### Managerial Implications

From a managerial perspective, BPMN offers significant potential in health care service planning [50,73,78,82,91-98], process optimization [47,53,83,85,91-98], and resource allocation [57,67,78,80,81,94,97]. By providing a standardized tool for modeling health care workflows, BPMN enables administrators and decision-makers to identify inefficiencies [43,44,48,57,61,81,91-95,97] and implement data-driven improvements [42,44,52,56,58,73,76,79,82,94,96]. However, managers must also carefully weigh the trade-offs between BPMN’s benefits and its limitations, particularly in highly complex health care settings, and ensure that the project is properly prepared, as implementing BPMN requires a significant investment in human resources and skills. The BPMN purpose-implementation-outcome model proposed in this review offers a structured framework to assess and refine BPMN transformation projects, ensuring that they align with clinical and organizational objectives.

### Future Research Directions

Several areas for future research could enhance our understanding of BPMN’s role in health care. Comparative studies directly evaluating BPMN against alternative modeling approaches could provide deeper insights into its strengths and weaknesses. More extensive, real-world implementation trials are needed to assess BPMN’s impact on patient outcomes and operational efficiency at scale. Additionally, further research into the integration of AI and process mining techniques could improve BPMN’s capabilities in predictive analytics and decision automation. Finally, investigating user-centric design approaches could help make BPMN more accessible and intuitive for health care professionals, facilitating broader adoption.

### Limitations

While this systematic review provides valuable insights, limitations should be acknowledged. The majority of the studies included are methodologically weak (75.4%), consisting of exploratory or pilot descriptive studies. Given the methodological weaknesses of existing studies and the limited number of large-scale BPMN implementation projects, the validity of the results presented remains questionable. Indeed, most studies focused on prototypes [42,46,49,51,70,73,81,88], small-scale descriptive projects [43,45,50,53,57,62,63,77,80,82-85,91-98], or theoretical explorations [41,44,55-57,59,66,68,69], underscoring the need for further real-world research. Furthermore, the concentration of studies included in European countries (68.9%) is a key factor limiting the generalizability of our results.

### Conclusions

This systematic review highlights BPMN’s potential as a valuable tool for modeling patient health care trajectories in a managerial approach to transforming practices. Its ability to visualize and optimize processes makes it a promising tool for improving clinical and operational efficiency. However, trade-offs between benefits and limits of BPMN characterize its implementation in patient health care trajectories, giving rise to opportunities for the development and integration of new tools and extensions to handle complexity and real-time data integration and to optimize outcomes. However, it is important to note that the methodological weaknesses of the studies and the lack of large-scale research projects mean that these results cannot be generalized. Future advancements, including the development of more sophisticated BPMN extensions, integration with AI, and improved interoperability with health care IT systems, will be crucial in realizing BPMN’s full potential. We propose a framework linking purpose to outcomes through richly characterized implementation domains, which could help managers to better specify their BPMN transformation projects and facilitate evaluation of their effectiveness. Future research could address the compatibility of systems in the hospital environment and emphasize the importance of considering the social dimension inherent in any change in professional and organizational practices.

## Supplementary material

10.2196/78506 Multimedia Appendix 1 Databases search strategy.

10.2196/78506 Multimedia Appendix 2 Extraction table (Business Process Modeling Notation).

10.2196/78506 Multimedia Appendix 3 Completed Mixed Method Appraisal Tool checklist.

10.2196/78506 Multimedia Appendix 4 Distribution of subthemes.

10.2196/78506 Checklist 1 Completed PRISMA 2020 checklist.

## Abbreviations

AI: artificial intelligence
BPMN: Business Process Modeling Notation
CFIR: Consolidated Framework for Implementation Research
CMMN: Case Management Model Notation
CPG: clinical practice guideline
DMN: Decision Modeling Notation
EHR: electronic health record
FHIR: Fast Healthcare Interoperability Resource
HL7: Health Level Seven
MMAT: Mixed Methods Appraisal Tool
PICOTS: Population, Intervention, Comparator, Outcomes, Timing, Settings
PRISMA: Preferred Reporting Items for Systematic Reviews and Meta-Analyses
