# Supplementary material for: The Implementation of a Business Process Model and Notation for Modeling Patient Health Care Trajectories: Systematic Review
Source: J Med Internet Res. 2026 Jun 9;28:e78506. doi: 10.2196/78506 (PMC13249068; doi:10.2196/78506)
Supplement: Multimedia Appendix 1 [file jmir-v28-e78506-s001.docx]

**The Implementation of Business Process Model and Notation (BPMN) for Modeling Patient Healthcare Trajectories: A Systematic Review**

Jean-Baptiste Gartner^a,b,c,d,e,*^ ^[0000-0001-5907-6112]^, Paolo Landa^f,b,c [0000-0001-6532-6747]^, Matthew T. Haren^a,b [0000-0003-2464-4364]^, Célia Lemaire^g,h [0000-0003-4576-0516]^, Elena Tanfani^i [0000-0002-4261-4495]^, Catherine Paquet^j,b,c [0000-0002-6877-7903]^, Frédéric Bergeron^k [0000-0003-0978-7420]^, André Côté^a,b,c,d,e,l [0000-0002-0748-594X]^

***Corresponding Author**

Jean-Baptiste Gartner,

Département de management, Faculté des sciences de l’administration, Université Laval

2325 rue de la Terrasse, Québec QC G1V 0A6‎, Canada

Email: [jean-baptiste.gartner@fsa.ulaval.ca](mailto:jean-baptiste.gartner@fsa.ulaval.ca)

**Author’s Affiliation**

^a^Département de management, Faculté des sciences de l’administration, Université Laval, Québec, QC, Canada.

^b^Centre de recherche en gestion des services de santé, Université Laval, Québec, QC, Canada.

^c^Centre de recherche du CHU de Québec - Université Laval, Québec, QC, Canada.

^d^VITAM, Centre de recherche en santé durable, Université Laval, Québec, QC, Canada.

^e^Centre de recherche du CISSS de Chaudière-Appalaches, Lévis, QC, Canada.

^f^Département d'opérations et systèmes de décision, Faculté des sciences de l’administration, Université Laval, Québec, QC, Canada.

^g^iaelyon School of Management, Université Lyon 3, Lyon, France.

^h^Institut Universitaire de France, Paris, France.

^i^Department of Economics, University of Genova, Genova, Italy.

^j^Département de Marketing, Faculté des sciences de l’administration, Université Laval, Québec, QC, Canada.

^k^Bibliothèque-Direction des services-conseils, Université Laval, Québec, QC, Canada.

^l^Centre de recherche de l'Institut Universitaire de Cardio-Pneumologie de Québec - Université Laval, QC, Canada.

**Databases search strategy**

**PubMed**

**Date of the original search:** 28-05-2020

**Subsequent update dates:** 05-10-2022; 03-10-2023; 05-01-2026

**Database limit:** only publications from 2004 to present have been considered.

| **Concepts** | **#** | **Search strategy** | **Results** |
| --- | --- | --- | --- |
| Business Process Modeling | 1 | Business Process Model*[TIAB] OR BPMN[OT] OR BPM[OT] OR Business Process Model*[OT] OR Business Process Method*[TIAB] OR Model* Business Process[TIAB] |  |
| Publications between 2004-01-01 to 28-05-2020 | 2 | #1 AND 2004:2020[dp] | 238 |
| Publications between 2020-05-29 to 2022-10-05 | 3 | #1 AND 2020/05/29:2022/10/05[dp] | 120 |
| Publications between 2022-10-06 to 2023-10-03 | 4 | #1 AND 2022/10/06:2023/10/03[dp] | 52 |
| Publications between 2023-10-03 to 2026-01-05 | 5 | #1 AND (2023/10/04:2026/01/05[edat] OR 2023/10/04:2026/01/05[crdt]) | 65 |
| **Total number of results** | 6 |  | 475 |

**Embase (Embase.com)**

**Date of the original search:** 28-05-2020

**Subsequent update dates:** 05-10-2022; 03-10-2023; 05-01-2026

**Database limit:** only publications from 2004 to present have been considered; and only Embase database results & prepublications have been considered.

| **Concepts** | **#** | **Search strategy** | **Results** |
| --- | --- | --- | --- |
| Business Process Modeling | 1 | "Business Process Model*":ti,ab,kw OR "Business Process Method*":ti,ab,kw OR "Model* Business Process":ti,ab,kw OR BPMN:kw OR BPM:kw |  |
| Embase & prepublication results only | 2 | #1 AND [embase]/lim NOT ([embase]/lim AND [medline]/lim) |  |
| Publications between 2004-01-01 to 28-05-2020 | 3 | #2 AND [2004-2020]/py | 43 |
| Publications between 2020-05-29 to 2022-10-05 | 4 | #2 AND [29-05-2020]/sd | 14 |
| Publications between 2022-10-06 to 2023-10-03 | 5 | #2 AND [06-10-2022]/sd | 12 |
| Publications between 2023-10-03 to 2026-01-05 | 6 | #2 AND [04-10-2023]/sd | 30 |
| **Total number of results** | 7 |  | 99 |

**CINAHL (EBSCO)**

**Date of the original search:** 28-05-2020

**Subsequent update dates:** 05-10-2022; 03-10-2023; 05-01-2026

**Database limit:** only publications from 2004 to present have been considered.

| **Concepts** | **#** | **Search strategy** | **Results** |
| --- | --- | --- | --- |
| Business Process Modeling | 1 | TI "Business Process Model*" OR TI "Business Process Method*" OR AB "Business Process Model*" OR AB "Business Process Method*" OR AB "Model* Business Process" |  |
| Publications between 2004-01-01 to 28-05-2020 | 2 | S1 AND DT 20040101-20201231 | 43 |
| Publications between 2020-05-29 to 2022-10-05 | 3 | S1 AND EM 20200529-20221005 | 8 |
| Publications between 2022-10-06 to 2023-10-03 | 4 | S1 AND EM 20221006-20231003 | 4 |
| Publications between 2023-10-03 to 2026-01-05 | 5 | S1 AND EM 20231004-20260105 | 1 |
| **Total number of results** | 6 |  | 56 |

**Web of Science**

**Date of the original search:** 28-05-2020

**Subsequent update dates:** 05-10-2022; 03-10-2023; 05-01-2026

**Database limit:** only publications from 2004 to present have been considered.

| **Concepts** | **#** | **Search strategy** | **Results** |
| --- | --- | --- | --- |
| Business Process Modeling | 1 | TS=("Business Process Model*") OR TS=("Business Process Method*") OR TS=("Model* Business Process") |  |
| Healthcare management | 2 | TS=(delivery NEAR/3 care) OR TS=(delivery NEAR/3 "Health service") OR TS=("health care") OR TS=(Healthcare) OR TS=(Health NEAR/3 quality) OR TS=((Clinical OR Critical) NEAR/3 path*) OR TS=((Clinical OR medical) NEAR/3 Processes) OR TS=("Patient Journey") |  |
| Combination of concepts | 3 | #1 AND #2 |  |
| Publications between 2004-01-01 to 28-05-2020 | 4 | #3 AND PY=(2004-2020) | 152 |
| Publications between 2020-05-29 to 2022-10-05 | 5 | #3 *with (database date limit between 20200529-20221005)* | 22 |
| Publications between 2022-10-06 to 2023-10-03 | 6 | #3 with (database date limit between 20221006-20231003) | 13 |
| Publications between 2023-10-03 to 2026-01-05 | 7 | #3 AND LD=(2023-10-04/2026-01-05) | 31 |
| **Total number of results** | 8 |  | 218 |

**Academic Search Premier**

**Date of the original search:** 28-05-2020

**Subsequent update dates:** 05-10-2022; 03-10-2023; 05-01-2026

**Database limit:** only publications from 2004 to present have been considered.

| **Concepts** | **#** | **Search strategy** | **Results** |
| --- | --- | --- | --- |
| Business Process Modeling | 1 | TI "Business Process Model*" OR TI "Business Process Method*" OR TI "Model* Business Process" OR AB "Business Process Model*" OR AB "Business Process Method*" OR AB "Model* Business Process" OR KW "Business Process Model*" |  |
| Healthcare management | 2 | TI (delivery N3 care) OR AB (delivery N3 care) OR KW (delivery N3 care) OR TI (delivery N3 "Health service") OR AB (delivery N3 "Health service") OR KW (delivery N3 "Health service") OR TI ("health care") OR AB ("health care")  OR KW ("health care") OR TI (Healthcare) OR AB (Healthcare) OR KW (Health N3 quality) OR TI ((Clinical OR Critical) N3 path*) OR AB ((Clinical OR Critical) N3 path*) OR KW ((Clinical OR Critical) N3 path*) OR TI ((Clinical OR medical) N3 processes) OR AB ((Clinical OR medical) N3 processes) OR KW ((Clinical OR medical) N3 processes) OR TI ("Patient Journey") OR AB ("Patient Journey") OR KW ("Patient Journey") OR DE "MEDICAL care" OR DE "HEALTH services accessibility" OR DE "MEDICAL quality control" OR DE "HEALTH outcome assessment" |  |
| Combination of concepts | 3 | S1 AND S2 |  |
| Publications between 2004-01-01 to 28-05-2020 | 4 | S3 AND DT 20040101-20201231 | 26 |
| Publications between 2020-05-29 to 2022-10-05 | 5 | S3 AND DT 20200529-20221005 | 5 |
| Publications between 2022-10-06 to 2023-10-03 | 6 | S3 AND DT 20221006-20231003 | 4 |
| Publications between 2023-10-03 to 2026-01-05 | 7 | S3 AND DT 20231004-20260105 | 85 |
| **Total number of results** | 8 |  | 120 |

**ABI/inform (ProQuest)**

**Date of the original search:** 28-05-2020

**Subsequent update dates:** 05-10-2022; 03-10-2023; 05-01-2026

**Database limit:** only publications from 2004 to present have been considered.

| **Concepts** | **#** | **Search strategy** | **Results** |
| --- | --- | --- | --- |
| Business Process Modeling | 1 | AB,TI("Business Process Model*") OR AB,TI("Business Process Method*") OR AB,TI("Model* Business Process") |  |
| Healthcare management | 2 | AB,TI(delivery NEAR/3 care) OR AB,TI(delivery NEAR/3 "Health service") OR AB,TI("health care") OR AB,TI(Healthcare) OR AB,TI(Health NEAR/3 quality) OR AB,TI((Clinical OR Critical) NEAR/3 path*) OR AB,TI((Clinical OR medical) NEAR/3 processes) OR AB,TI("Patient Journey") OR SU("Health care delivery") OR SU(Quality of care) OR SU(Health care) |  |
| Combination of concepts | 3 | [S1] AND [S2] |  |
| Publications between 2004-01-01 to 28-05-2020 | 4 | 3 AND YR(2004-2020) | 17 |
| Publications between 2020-05-29 to 2022-10-05 | 5 | 3 AND YR(2020-2022) | 0 |
| Publications between 2022-10-06 to 2023-10-03 | 6 | 3 AND YR(2022-2023) | 0 |
| Publications between 2023-10-03 to 2026-01-05 | 7 | [S3] AND PD(20231004-20260105) | 6 |
| **Total number of results** | 8 |  | 23 |

**ScienceDirect**

**Date of the original search:** 28-05-2020

**Subsequent update dates:** 05-10-2022; 03-10-2023; 05-01-2026

**Database limit:** only publications from 2004 to present have been considered.

| **Search strategy** | | **Results** |
| --- | --- | --- |
| Original search strategy | Anywhere: ("Business Process Model" OR "Business Process Modeling" OR "Business Process Models") AND Title-abstract-authorkeywords: ("Health service" OR "health care" OR "Healthcare" OR "MEDICAL care" OR "clinical pathway" OR "medical pathway" OR "Patient Journey" OR "Quality of care") | 106 |
| First updated search strategy | Anywhere: ("Business Process Model" OR "Business Process Modeling" OR "Business Process Models") AND Title-abstract-authorkeywords: ("Health service" OR "health care" OR "Healthcare" OR "MEDICAL care" OR "clinical pathway" OR "medical pathway" OR "Patient Journey" OR "Quality of care") | 37 |
| Second updated search strategy | Anywhere: ("Business Process Model" OR "Business Process Modeling" OR "Business Process Models") AND Title-abstract-authorkeywords: ("Health service" OR "health care" OR "Healthcare" OR "MEDICAL care" OR "clinical pathway" OR "medical pathway" OR "Patient Journey" OR "Quality of care") | 26 |
| Third updated search strategy | Anywhere: ("Business Process Model" OR "Business Process Modeling" OR "Business Process Models") AND Title-abstract-authorkeywords: ("Health service" OR "health care" OR "Healthcare" OR "MEDICAL care" OR "clinical pathway" OR "medical pathway" OR "Patient Journey" OR "Quality of care") | 71 |
| **Total number of results** | 240 | |

**Google Scholar (https://harzing.com/resources/publish-or-perish)**

**Date of the search:** 03-10-2023

**Database limit:** only up to the 30 first results per string have been considered; publications between 2022 to 2023 limit has been apply; citations and patents options have been removed.

| **#** | **Search** | **# Results screened** |
| --- | --- | --- |
| 1 | ("Business Process Model" OR "Business Process Modelling" OR "Business Process Modeling") AND "patient journey" | 30 |
| 2 | ("Business Process Model" OR "Business Process Modelling" OR "Business Process Modeling" OR "Business Process management") AND "clinical pathways" | 30 |
| 3 | ("Business Process Model" OR "Business Process Modelling" OR "Business Process Modeling" OR "Business Process management") AND "health care" | 30 |
| 4 | ("Business Process Model" OR "Business Process Modelling" OR "Business Process Modeling" OR "Business Process management") AND healthcare | 30 |
| 5 | ("Business Process Model" OR "Business Process Modelling" OR "Business Process Modeling" OR "Business Process management") AND "patient care" | 30 |
| 6 | ("Business Process Model" OR "Business Process Modelling" OR "Business Process Modeling") AND "Health service" | 30 |
|  | **Total number of results** | **180** |

**Google Scholar (https://harzing.com/resources/publish-or-perish)**

**Date of the search:** 05-01-2026

**Database limit:** only up to the 30 first results per string have been considered; publications between 2023 to 2026 limit has been apply; citations and patents options have been removed.

| **#** | **Search** | **# Results screened** |
| --- | --- | --- |
| 1 | ("Business Process Model" OR "Business Process Modelling" OR "Business Process Modeling") AND "patient journey" | 30 |
| 2 | ("Business Process Model" OR "Business Process Modelling" OR "Business Process Modeling" OR "Business Process management") AND "clinical pathways" | 30 |
| 3 | ("Business Process Model" OR "Business Process Modelling" OR "Business Process Modeling" OR "Business Process management") AND "health care" | 30 |
| 4 | ("Business Process Model" OR "Business Process Modelling" OR "Business Process Modeling" OR "Business Process management") AND healthcare | 30 |
| 5 | ("Business Process Model" OR "Business Process Modelling" OR "Business Process Modeling" OR "Business Process management") AND "patient care" | 30 |
| 6 | ("Business Process Model" OR "Business Process Modelling" OR "Business Process Modeling") AND "Health service" | 30 |
|  | **Total number of results** | **180** |
