# Supplementary material for: The Implementation of a Business Process Model and Notation for Modeling Patient Health Care Trajectories: Systematic Review
Source: J Med Internet Res. 2026 Jun 9;28:e78506. doi: 10.2196/78506 (PMC13249068; doi:10.2196/78506)
Supplement: Multimedia Appendix 3 [file jmir-v28-e78506-s003.docx]

**The Implementation of Business Process Model and Notation (BPMN) for Modeling Patient Healthcare Trajectories: A Systematic Review**

Jean-Baptiste Gartner^1,2,3,4,5,*^ ^[0000-0001-5907-6112]^, Paolo Landa^6,2,3 [0000-0001-6532-6747]^, Matthew T. Haren^1,2 [0000-0003-2464-4364]^, Célia Lemaire^7,8 [0000-0003-4576-0516]^, Elena Tanfani^9 [0000-0002-4261-4495]^, Catherine Paquet^10,2,3 [0000-0002-6877-7903]^, Frédéric Bergeron^11 [0000-0003-0978-7420]^, André Côté^1,2,3,4,5,12 [0000-0002-0748-594X]^

***Corresponding Author**

Jean-Baptiste Gartner,

Département de management, Faculté des sciences de l’administration, Université Laval

2325 rue de la Terrasse, Québec QC G1V 0A6‎, Canada

Email: [jean-baptiste.gartner@fsa.ulaval.ca](mailto:jean-baptiste.gartner@fsa.ulaval.ca)

**Author’s Affiliation**

^1^Département de management, Faculté des sciences de l’administration, Université Laval, Québec, QC, Canada.

^2^Centre de recherche en gestion des services de santé, Université Laval, Québec, QC, Canada.

^3^Centre de recherche du CHU de Québec - Université Laval, Québec, QC, Canada.

^4^VITAM, Centre de recherche en santé durable, Université Laval, Québec, QC, Canada.

^5^Centre de recherche du CISSS de Chaudière-Appalaches, Lévis, QC, Canada.

^6^Département d'opérations et systèmes de décision, Faculté des sciences de l’administration, Université Laval, Québec, QC, Canada.

^7^iaelyon School of Management, Université Lyon 3, Lyon, France.

^8^Institut Universitaire de France, Paris, France.

^9^Department of Economics, University of Genova, Genova, Italy.

^10^Département de Marketing, Faculté des sciences de l’administration, Université Laval, Québec, QC, Canada.

^11^Bibliothèque-Direction des services-conseils, Université Laval, Québec, QC, Canada.

^12^Centre de recherche de l'Institut Universitaire de Cardio-Pneumologie de Québec - Université Laval, QC, Canada.

**Completed Mixed Method Appraisal Tool (MMAT) checklist**

| **MMAT 2018 item checklist** | | | | | | | | | | | | | | | | | | | | | | | | | | | | | | |
| --- | --- | --- | --- | --- | --- | --- | --- | --- | --- | --- | --- | --- | --- | --- | --- | --- | --- | --- | --- | --- | --- | --- | --- | --- | --- | --- | --- | --- | --- | --- |
|  |  |  |  | **SCREENING QUESTIONS** | | **1. QUALITATIVE STUDIES** | | | | | **2. RANDOMIZED CONTROLLED TRIALS** | | | | | **3. NON-RANDOMIZED STUDIES** | | | | | **4. QUANTITATIVE DESCRIPTIVE STUDIES** | | | | | **5. MIXED METHODS STUDIES** | | | | |
| **RefID** | **First author** | **Year** | **Title** | S1. Are there clear research questions? | S2. Do the collected data allow to address the research questions? | 1.1. Is the qualitative approach appropriate to answer the research question? | 1.2. Are the qualitative data collection methods adequate to address the research question? | 1.3. Are the findings adequately derived from the data? | 1.4. Is the interpretation of results sufficiently substantiated by data? | 1.5. Is there coherence between qualitative data sources, collection, analysis and interpretation? | 2.1. Is randomization appropriately performed? | 2.2. Are the groups comparable at baseline? | 2.3. Are there complete outcome data? | 2.4. Are outcome assessors blinded to the intervention provided? | 2.5 Did the participants adhere to the assigned intervention? | 3.1. Are the participants representative of the target population? | 3.2. Are measurements appropriate regarding both the outcome and intervention (or exposure)? | 3.3. Are there complete outcome data? | 3.4. Are the confounders accounted for in the design and analysis? | 3.5. During the study period, is the intervention administered (or exposure occurred) as intended? | 4.1. Is the sampling strategy relevant to address the research question? | 4.2. Is the sample representative of the target population? | 4.3. Are the measurements appropriate? | 4.4. Is the risk of nonresponse bias low? | 4.5. Is the statistical analysis appropriate to answer the research question? | 5.1. Is there an adequate rationale for using a mixed methods design to address the research question? | 5.2. Are the different components of the study effectively integrated to answer the research question? | 5.3. Are the outputs of the integration of qualitative and quantitative components adequately interpreted? | 5.4. Are divergences and inconsistencies between quantitative and qualitative results adequately addressed? | 5.5. Do the different components of the study adhere to the quality criteria of each tradition of the methods involved? |
| 1 | #592 – Ajmi 2015 | 2015 | Mapping patient path in the Pediatric Emergency Department: A workflow model driven approach | **No** | **No** | **Can't tell** | **Can't tell** | **Can't tell** | **Can't tell** | **Can't tell** |  |  |  |  |  |  |  |  |  |  |  |  |  |  |  |  |  |  |  |  |
| 2 | #581 – Ateetanan 2017 | 2017 | From Service Design to Enterprise Architecture: The Alignment of Service Blueprint and Business Architecture with Business Process Model and Notation | **No** | **No** | **Can't tell** | **Can't tell** | **Can't tell** | **Can't tell** | **Can't tell** |  |  |  |  |  |  |  |  |  |  |  |  |  |  |  |  |  |  |  |  |
| 3 | #572 – Barbagallo 2015 | 2015 | Optimization and planning of operating theatre activities: an original definition of pathways and process modeling | **Yes** | **Yes** | **Can't tell** | **Can't tell** | **Can't tell** | **Can't tell** | **Can't tell** |  |  |  |  |  |  |  |  |  |  | **Yes** | **Yes** | **Yes** | **Yes** | **Yes** | **Yes** | **Yes** | **Yes** | **Can't tell** | **Can't tell** |
| 4 | #448 – Bazhenova 2019 | 2019 | From BPMN process models to DMN decision models | **Yes** | **Yes** | **Can't tell** | **Can't tell** | **Can't tell** | **Can't tell** | **Can't tell** |  |  |  |  |  |  |  |  |  |  | **Yes** | **Yes** | **Yes** | **Yes** | **Yes** | **Yes** | **Yes** | **Yes** | **Yes** | **Can't tell** |
| 5 | #441 – BenHassen 2017 | 2017 | BPMN4KM: Design and Implementation of a BPMN Extension for Modeling the Knowledge Perspective of Sensitive Business Processes | **Yes** | **Can't tell** |  |  |  |  |  |  |  |  |  |  |  |  |  |  |  |  |  |  |  |  |  |  |  |  |  |
| 6 | #428 – Bisogno 2016 | 2016 | Combining modelling and simulation approaches How to measure performance of business processes | **Yes** | **Yes** |  |  |  |  |  |  |  |  |  |  |  |  |  |  |  | **Yes** | **Yes** | **Yes** | **Yes** | **Yes** |  |  |  |  |  |
| 7 | #416 – Braun 2016 | 2016 | BPMN4CP Revised - Extending BPMN for Multi-Perspective Modeling of Clinical Pathways | **No** | **Can't tell** |  |  |  |  |  |  |  |  |  |  |  |  |  |  |  |  |  |  |  |  |  |  |  |  |  |
| 8 | #417 – Braun 2014 | 2014 | BPMN4CP: Design and Implementation of a BPMN Extension for Clinical Pathways | **No** | **Can't tell** |  |  |  |  |  |  |  |  |  |  |  |  |  |  |  |  |  |  |  |  |  |  |  |  |  |
| 9 | #404 – Çatal 2017 | 2017 | Supporting process execution by interdisciplinary healthcare teams: Middleware design for IBM BPM | **No** | **Can't tell** |  |  |  |  |  |  |  |  |  |  |  |  |  |  |  |  |  |  |  |  |  |  |  |  |  |
| 10 | #384 – Combi 2016 | 2016 | Seamless Design of Decision-Intensive Care Pathways | **No** | **Can't tell** |  |  |  |  |  |  |  |  |  |  |  |  |  |  |  |  |  |  |  |  |  |  |  |  |  |
| 11 | #383 – Combi 2017 | 2017 | Towards Dynamic Duration Constraints for Therapy and Monitoring Tasks | **No** | **Can't tell** |  |  |  |  |  |  |  |  |  |  |  |  |  |  |  |  |  |  |  |  |  |  |  |  |  |
| 12 | #377 – Crisan-Vida 2013 | 2013 | ICT Solution for Assisted Diagnosis Based on Monitoring in Cardiology Departments | **No** | **Can't tell** |  |  |  |  |  |  |  |  |  |  |  |  |  |  |  |  |  |  |  |  |  |  |  |  |  |
| 13 | #373 – Cutting 2015 | 2015 | Using Workflow Modeling to Identify Areas to Improve Genetic Test Processes in the University of Maryland Translational Pharmacogenomics Project | **Yes** | **Yes** | **Yes** | **Yes** | **Yes** | **Yes** | **Yes** |  |  |  |  |  |  |  |  |  |  |  |  |  |  |  |  |  |  |  |  |
| 14 | #361 – deBruin 2018 | 2018 | Separating Business Logic from Medical Knowledge in Digital Clinical Workflows Using Business Process Model and Notation and Arden Syntax | **No** | **Can't tell** |  |  |  |  |  |  |  |  |  |  |  |  |  |  |  |  |  |  |  |  |  |  |  |  |  |
| 15 | #339 – Eklund 2009 | 2009 | BPMN and its Semantics for Information Management in Emergency Care | **No** | **No** |  |  |  |  |  |  |  |  |  |  |  |  |  |  |  |  |  |  |  |  |  |  |  |  |  |
| 16 | #322 – Fox 2008 | 2008 | From guidelines to careflows: modelling and supporting complex clinical processes | **No** | **No** |  |  |  |  |  |  |  |  |  |  |  |  |  |  |  |  |  |  |  |  |  |  |  |  |  |
| 17 | #297 – Haouari 2017 | 2017 | Quality Assessment of an Emergency Care Process Model based on Static and Dynamic Metrics | **No** | **No** |  |  |  |  |  |  |  |  |  |  |  |  |  |  |  |  |  |  |  |  |  |  |  |  |  |
| 18 | #283 – Hewelt 2015 | 2015 | Recommendations for Medical Treatment Processes: The PIGS Approach | **No** | **No** |  |  |  |  |  |  |  |  |  |  |  |  |  |  |  |  |  |  |  |  |  |  |  |  |  |
| 19 | #262 – Ilahi 2016 | 2016 | Similarity Based Approach for Comparing Home Healthcare Processes Models in Portugal | **Yes** | **Yes** | **Yes** | **Yes** | **Yes** | **Yes** | **Yes** |  |  |  |  |  | **Yes** | **Yes** | **Yes** | **Yes** | **Yes** |  |  |  |  |  | **Yes** | **Yes** | **Yes** | **Yes** | **Yes** |
| 20 | #252 – Jimenez-Molina 2018 | 2018 | ProFUSO: Business process and ontology-based framework to develop ubiquitous computing support systems for chronic patients' management | **Yes** | **Yes** |  |  |  |  |  |  |  |  |  |  |  |  |  |  |  | **Can't tell** | **Can't tell** | **Yes** | **Can't tell** | **Yes** |  |  |  |  |  |
| 21 | #225 – Kopecky 2020 | 2020 | The Business Process Model and Notation Used for the Representation of Alzheimer's Disease Patients Care Process | **No** | **No** |  |  |  |  |  |  |  |  |  |  |  |  |  |  |  |  |  |  |  |  |  |  |  |  |  |
| 22 | #216 – Lamine 2014 | 2014 | Ontology-Based Workflow Design for the Coordination of Homecare Interventions | **No** | **No** |  |  |  |  |  |  |  |  |  |  |  |  |  |  |  |  |  |  |  |  |  |  |  |  |  |
| 23 | #119 – Nan 2017 | 2017 | A meta-model for computer executable dynamic clinical safety checklists | **Yes** | **No** |  |  |  |  |  |  |  |  |  |  |  |  |  |  |  |  |  |  |  |  |  |  |  |  |  |
| 24 | #115 – Neumann 2019 | 2019 | Extending BPMN 2.0 for intraoperative workflow modeling with IEEE 11073 SDC for description and orchestration of interoperable, networked medical devices | **No** | **No** |  |  |  |  |  |  |  |  |  |  |  |  |  |  |  |  |  |  |  |  |  |  |  |  |  |
| 25 | #552 – Penteado 2015 | 2015 | Kidney Transplantation Process in Brazil Represented in Business Process Modeling Notation | **No** | **No** |  |  |  |  |  |  |  |  |  |  |  |  |  |  |  |  |  |  |  |  |  |  |  |  |  |
| 26 | #542 – Poulymenopoulou 2014 | 2014 | Adaptive healthcare processes for personalized emergency clinical pathways | **No** | **No** |  |  |  |  |  |  |  |  |  |  |  |  |  |  |  |  |  |  |  |  |  |  |  |  |  |
| 27 | #524 – Ramos-Merino 2018 | 2018 | A BPMN Based Notation for the Representation of Workflows in Hospital Protocols | **Yes** | **Yes** |  |  |  |  |  |  |  |  |  |  |  |  |  |  |  | **Yes** | **Can't tell** | **Yes** | **Can't tell** | **Yes** |  |  |  |  |  |
| 28 | #507 – Rodriguez-Loya 2014 | 2014 | A service oriented approach for guidelines-based clinical decision support using BPMN | **No** | **No** |  |  |  |  |  |  |  |  |  |  |  |  |  |  |  |  |  |  |  |  |  |  |  |  |  |
| 29 | #490 – Ruiz-Fernández 2017 | 2017 | Empowerment of Patients with Hypertension through BPM, IoT and Remote Sensing | **Yes** | **No** |  |  |  |  |  |  |  |  |  |  |  |  |  |  |  |  |  |  |  |  |  |  |  |  |  |
| 30 | #487 – Russo 2015 | 2015 | A Business Process Model for Integrated Home Care | **No** | **No** |  |  |  |  |  |  |  |  |  |  |  |  |  |  |  |  |  |  |  |  |  |  |  |  |  |
| 31 | #475 – Scheuerlein 2012 | 2012 | New methods for clinical pathways-Business Process Modeling Notation (BPMN) and Tangible Business Process Modeling (t.BPM) | **Yes** | **Yes** | **Yes** | **Yes** | **Yes** | **Yes** | **Can't tell** |  |  |  |  |  |  |  |  |  |  |  |  |  |  |  |  |  |  |  |  |
| 32 | #452 – Sooter 2019 | 2019 | Modeling a Clinical Pathway for Contraception | **No** | **No** |  |  |  |  |  |  |  |  |  |  |  |  |  |  |  |  |  |  |  |  |  |  |  |  |  |
| 33 | #77 – Svagard 2009 | 2009 | Using Business Process Modelling to Model Integrated Care Processes: Experiences from a European Project | **No** | **No** |  |  |  |  |  |  |  |  |  |  |  |  |  |  |  |  |  |  |  |  |  |  |  |  |  |
| 34 | #63 – Tomaskova 2019 | 2019 | Process Cost Management of Alzheimer's Disease | **Yes** | **Yes** | **Yes** | **Yes** | **Yes** | **Yes** | **Yes** |  |  |  |  |  |  |  |  |  |  | **Yes** | **Yes** | **Yes** | **Yes** | **Yes** | **Yes** | **Yes** | **Yes** | **Yes** | **Can't tell** |
| 35 | #50 – Vandborg 2012 | 2012 | A new method for analyzing diagnostic delay in gynecological cancer | **Yes** | **Yes** | **Yes** | **Yes** | **Yes** | **Yes** | **Yes** |  |  |  |  |  |  |  |  |  |  | **Yes** | **Can't tell** | **Yes** | **Yes** | **Yes** | **Yes** | **Yes** | **Yes** | **Yes** | **Yes** |
| 36 | #49 – Vinci 2018 | 2018 | The process of outpatient care of children and adolescents in a tertiary-level hospital specializing in pediatrics: A case study focused on identifying opportunities for improvement with the aid of modeling using BPMN | **Yes** | **Yes** | **Yes** | **Yes** | **Yes** | **Yes** | **Yes** |  |  |  |  |  |  |  |  |  |  |  |  |  |  |  |  |  |  |  |  |
| 37 | #37 – Weber 2018 | 2018 | Automated conflict detection between medical care pathways | **Yes** | **Yes** | **Yes** | **Yes** | **Yes** | **Yes** | **Can't tell** |  |  |  |  |  | **Can't tell** | **Yes** | **Yes** | **Can't tell** | **Yes** |  |  |  |  |  | **Yes** | **Yes** | **Yes** | **Yes** | **Yes** |
| 38 | #29 – Wiemuth 2017 | 2017 | Application fields for the new Object Management Group (OMG) Standards Case Management Model and Notation (CMMN) and Decision Management Notation (DMN) in the perioperative field | **No** | **No** |  |  |  |  |  |  |  |  |  |  |  |  |  |  |  |  |  |  |  |  |  |  |  |  |  |
| 39 | #6 – Zerbato 2015 | 2015 | BPMN-based Representation and Comparison of Clinical Pathways for Catheter-related Bloodstream Infections | **No** | **No** |  |  |  |  |  |  |  |  |  |  |  |  |  |  |  |  |  |  |  |  |  |  |  |  |  |
| 40 | #621 – Rodrigues 2021 | 2021 | Process modeling: technological innovation to control the risk for perioperative positioning injury | **No** | **No** |  |  |  |  |  |  |  |  |  |  |  |  |  |  |  |  |  |  |  |  |  |  |  |  |  |
| 41 | #630 – Aissaoui 2022 | 2022 | A BPMN-VSM based process analysis to improve the efficiency of multidisciplinary outpatient clinics | **Yes** | **Yes** | **Yes** | **Yes** | **Yes** | **Yes** | **Yes** |  |  |  |  |  |  |  |  |  |  |  |  |  |  |  |  |  |  |  |  |
| 42 | #796 – Szelagowski | 2021 | Proposal of BPMN extension with a view to effective modeling of clinical pathways | **No** | **No** |  |  |  |  |  |  |  |  |  |  |  |  |  |  |  |  |  |  |  |  |  |  |  |  |  |
| 43 | #586 – Alhaj 2017 | 2017 | Towards a model-based approach for developing and QA of online business processes | **No** | **No** | **Can't tell** | **Can't tell** | **Can't tell** | **Can't tell** | **Can't tell** |  |  |  |  |  |  |  |  |  |  |  |  |  |  |  |  |  |  |  |  |
| 44 | #829 – Yang 2021 | 2021 | On Treatment Patterns for Modeling Medical Treatment Processes in Clinical Practice Guidelines | **No** | **No** |  |  |  |  |  |  |  |  |  |  |  |  |  |  |  |  |  |  |  |  |  |  |  |  |  |
| 45 | #795 – Szelągowski 2021 | 2021 | BPM Support for Patient-Centred Clinical Pathways in Chronic Diseases | **No** | **No** |  |  |  |  |  |  |  |  |  |  |  |  |  |  |  |  |  |  |  |  |  |  |  |  |  |
| 46 | #652 – Bianchi 2021 | 2021 | Putting BPMN and DMN to Work: a Pediatric Surgery Case Study | **No** | **Can't tell** |  |  |  |  |  |  |  |  |  |  |  |  |  |  |  |  |  |  |  |  |  |  |  |  |  |
| 47 | #668 – deLima 2022 | 2021 | Mental health indicators in the hospitalization process in a Brazilian psychosocial care network | **No** | **No** |  |  |  |  |  |  |  |  |  |  |  |  |  |  |  |  |  |  |  |  |  |  |  |  |  |
| 48 | #645 – Beckmann 2022 | 2022 | Guideline-Based Context-Sensitive Decision Modeling for Melanoma Patients | **No** | **Can't tell** |  |  |  |  |  |  |  |  |  |  |  |  |  |  |  |  |  |  |  |  |  |  |  |  |  |
| 49 | #961 – Iglesias 2022 | 2022 | Handling Time Constraints in Infection Clinical Pathways Using openEHR TP | **No** | **No** |  |  |  |  |  |  |  |  |  |  |  |  |  |  |  |  |  |  |  |  |  |  |  |  |  |
| 50 | #988 – Kirisits 2023 | 2023 | Process-Modelling of Cross-Sector Healthcare Quality Indicators | **No** | **No** |  |  |  |  |  |  |  |  |  |  |  |  |  |  |  |  |  |  |  |  |  |  |  |  |  |
| 51 | #1012 – Litchfield 2022 | 2022 | Automated conflict resolution for patients with multiple morbidity being treated using more than one set of single condition clinical guidance: A case study | **Yes** | **Can't tell** |  |  |  |  |  |  |  |  |  |  |  |  |  |  |  |  |  |  |  |  |  |  |  |  |  |
| 52 | #991 – Kober 2023 | 2023 | Using BPMN for medical guidelines that integrate with FHIR-RDF | **No** | **No** |  |  |  |  |  |  |  |  |  |  |  |  |  |  |  |  |  |  |  |  |  |  |  |  |  |
| 53 | #1023 – Martínez-Salvador 2023 | 2023 | A model-driven transformation approach for the modelling of processes in clinical practice guidelines | **No** | **Can't tell** |  |  |  |  |  |  |  |  |  |  |  |  |  |  |  |  |  |  |  |  |  |  |  |  |  |
| 54 | #1457 – Rasooli 2024 | 2024 | BPM application in clinical process improvement: a women'hospital case study | **No** | **No** |  |  |  |  |  |  |  |  |  |  |  |  |  |  |  |  |  |  |  |  |  |  |  |  |  |
| 55 | #1473 – Rocco 2024 | 2024 | Improving care pathways through BPM and telemedicine: an Italian study | **Yes** | **No** |  |  |  |  |  |  |  |  |  |  |  |  |  |  |  |  |  |  |  |  |  |  |  |  |  |
| 56 | #1326 – Knight 2024 | 2024 | Visualizing Patient Pathways and Identifying Data Repositories in a UK Neurosciences Center: Exploratory Study | **No** | **No** |  |  |  |  |  |  |  |  |  |  |  |  |  |  |  |  |  |  |  |  |  |  |  |  |  |
| 57 | #1483 – Rosa 2024 | 2024 | Business Process Modelling to Optimise the Management of Surgeries of Acute Cholecystitis: A Case Study of an Italian Hospital Unit | **No** | **No** |  |  |  |  |  |  |  |  |  |  |  |  |  |  |  |  |  |  |  |  |  |  |  |  |  |
| 58 | #1212 – Calabrese 2024 | 2024 | Design and development of a digital diagnostic clinical pathway: evidence from an action research study | **Yes** | **Yes** | **Yes** | **Yes** | **Yes** | **Yes** | **Yes** |  |  |  |  |  |  |  |  |  |  | **Yes** | **Can't tell** | **Yes** | **Can't tell** | **Yes** | **Yes** | **Yes** | **Yes** | **Can't tell** | **Yes** |
| 59 | #1241 – DosSantosLeandro 2024 | 2024 | FHIR Implementation Guide for Stroke: A dual focus on the patient's clinical pathway and value-based healthcare | **No** | **No** |  |  |  |  |  |  |  |  |  |  |  |  |  |  |  |  |  |  |  |  |  |  |  |  |  |
| 60 | #1383 – Madan 2025 | 2025 | The National Health Service 2-week-wait skin cancer referral pathway: analysis and recommendations for process improvement | **No** | **No** |  |  |  |  |  |  |  |  |  |  |  |  |  |  |  |  |  |  |  |  |  |  |  |  |  |
| 61 | #1431 – Odeh 2025 | 2025 | Bridging the digital readiness gap in palliative home care: A process to data approach | **Yes** | **Yes** | **Yes** | **Yes** | **Yes** | **Yes** |  |  |  |  |  |  |  |  |  |  | **Yes** | **Can't tell** | **Yes** | **Can't tell** | **Yes** | **Yes** | **Yes** | **Yes** | **Yes** | **Yes** | **Yes** |
