# Supplementary material for: The Implementation of a Business Process Model and Notation for Modeling Patient Health Care Trajectories: Systematic Review
Source: J Med Internet Res. 2026 Jun 9;28:e78506. doi: 10.2196/78506 (PMC13249068; doi:10.2196/78506)
Supplement: Multimedia Appendix 4 [file jmir-v28-e78506-s004.docx]

**The Implementation of Business Process Model and Notation (BPMN) for Modeling Patient Healthcare Trajectories: A Systematic Review**

Jean-Baptiste Gartner^a,b,c,d,e,*^ ^[0000-0001-5907-6112]^, Paolo Landa^f,b,c [0000-0001-6532-6747]^, Matthew T. Haren^a,b [0000-0003-2464-4364]^, Célia Lemaire^g,h [0000-0003-4576-0516]^, Elena Tanfani^i [0000-0002-4261-4495]^, Catherine Paquet^j,b,c [0000-0002-6877-7903]^, Frédéric Bergeron^k [0000-0003-0978-7420]^, André Côté^a,b,c,d,e,l [0000-0002-0748-594X]^

***Corresponding Author**

Jean-Baptiste Gartner,

Département de management, Faculté des sciences de l’administration, Université Laval

2325 rue de la Terrasse, Québec QC G1V 0A6‎, Canada

Email: [jean-baptiste.gartner@fsa.ulaval.ca](mailto:jean-baptiste.gartner@fsa.ulaval.ca)

**Author’s Affiliation**

^a^Département de management, Faculté des sciences de l’administration, Université Laval, Québec, QC, Canada.

^b^Centre de recherche en gestion des services de santé, Université Laval, Québec, QC, Canada.

^c^Centre de recherche du CHU de Québec - Université Laval, Québec, QC, Canada.

^d^VITAM, Centre de recherche en santé durable, Université Laval, Québec, QC, Canada.

^e^Centre de recherche du CISSS de Chaudière-Appalaches, Lévis, QC, Canada.

^f^Département d'opérations et systèmes de décision, Faculté des sciences de l’administration, Université Laval, Québec, QC, Canada.

^g^iaelyon School of Management, Université Lyon 3, Lyon, France.

^h^Institut Universitaire de France, Paris, France.

^i^Department of Economics, University of Genova, Genova, Italy.

^j^Département de Marketing, Faculté des sciences de l’administration, Université Laval, Québec, QC, Canada.

^k^Bibliothèque-Direction des services-conseils, Université Laval, Québec, QC, Canada.

^l^Centre de recherche de l'Institut Universitaire de Cardio-Pneumologie de Québec - Université Laval, QC, Canada.

**Distribution of sub-themes within each dominant theme of each category**

**Table S1** Distribution of sub-themes within each dominant theme of each category (benefits, limits, opportunities, alternatives/threats). Data represent counts (%) of extractions pertaining to each theme and sub-theme.

| **Theme** | **Benefits** | **Limits** | **Opportunities** | **Alternatives / threats** |
| --- | --- | --- | --- | --- |
| *language utility* | *49* | *7* | *6* | *5* |
| language utility | 21 (42.9) | 2 (28.6) |  |  |
| preferred language | 8 (16.3) |  |  |  |
| standard language | 8 (16.3) |  |  |  |
| standardized | 7 (14.3) |  |  |  |
| cross-professional language | 3 (6.1) |  |  |  |
| semantics development | 1 (2.0) | 1 (14.3) | 3 (50.0) |  |
| process definition | 1 (2.0) |  | 2 (33.3) |  |
| semantics |  | 4 (57.1) |  |  |
| pathway classification |  |  | 1 (16.7) |  |
| process language |  |  |  | 5 (100.0) |
| *management utility* | *27* | *10* | *11* | *0* |
| performance monitoring | 9 (33.3) | 2 (20.0) |  |  |
| management function | 6 (22.2) | 1 (10.0) |  |  |
| process understanding | 5 (18.5) | 1 (10.0) |  |  |
| quality improvement | 3 (11.1) | 1 (10.0) | 2 (18.2) |  |
| activity identification | 2 (7.4) | 1 (10.0) |  |  |
| efficiency and impact evaluation | 2 (7.4) | 1 (10.0) |  |  |
| narrow scope of business model domains |  | 3 (30.0) |  |  |
| *comprehensiveness* | *18* | *18* | *7* | *2* |
| comprehensive | 9 (50.0) | 2 (11.1) | 1 (14.3) |  |
| detailed | 5 (27.8) |  |  |  |
| time dependencies | 2 (11.1) |  |  | 1 (50.0) |
| complete patient journey | 2 (11.1) |  |  |  |
| missing concept (timing) |  | 7 (38.9) | 1 (14.3) |  |
| insufficient model of unstructured process |  | 4 (22.2) |  | 1 (50.0) |
| missing concept (multi-perspectivity) |  | 2 (11.1) |  |  |
| attribute description |  | 1 (5.6) | 1 (14.3) |  |
| missing concept (checklists) |  | 1 (5.6) | 2 (28.6) |  |
| missing concept (colour indicators) |  | 1 (5.6) | 1 (14.3) |  |
| multi-perspectivity |  |  | 1 (14.3) |  |
| *clinical utility* | *19* | *9* | *20* | *0* |
| clinical effectiveness | 4 (21.1) |  |  |  |
| clinical guideline utility | 3 (15.8) | 1 (11.1) | 3 (15.0) |  |
| patient monitoring | 3 (15.8) |  | 2 (10.0) |  |
| clinical pathways | 2 (10.5) | 5 (55.6) | 4 (20.0) |  |
| clinical education | 2 (10.5) |  |  |  |
| clinical safety | 2 (10.5) |  |  |  |
| quality care | 2 (10.5) |  |  |  |
| clinical utility | 1 (5.3) | 1 (11.1) | 1 (5.0) |  |
| patient-as-partner |  | 1 (11.1) |  |  |
| clinical guideline integration |  | 1 (11.1) | 1 (5.0) |  |
| patient pathway navigation |  |  | 2 (10.0) |  |
| patient safety |  |  | 1 (5.0) |  |
| recommendations, standards, best practices |  |  | 1 (5.0) |  |
| clinical outcomes |  |  | 1 (5.0) |  |
| patient preferences |  |  | 1 (5.0) |  |
| ML and AI informed treatment |  |  | 1 (5.0) |  |
| treatment compliance |  |  | 1 (5.0) |  |
| limit insurance claims (certify correct treatment) |  |  | 1 (5.0) |  |
| *user experience* | *19* | *8* | *3* | *2* |
| cross-professional language | 6 (31.6) |  |  |  |
| recognition | 6 (31.6) |  |  |  |
| simplicity | 4 (21.1) | 2 (25.0) |  |  |
| useability | 2 (10.5) |  | 1 (33.3) | 2 (100.0) |
| training requirement | 1 (5.3) | 1 (12.5) |  |  |
| lacks application guidance |  | 3 (37.5) |  |  |
| user interaction required |  | 1 (12.5) |  |  |
| complexity |  | 1 (12.5) |  |  |
| modular construction system |  |  | 2 (66.6) |  |
| *visualization* | *20* | *0* | *1* | *2* |
| visualization | 19 (95.0) |  | 1 (100.0) | 2 (100.0) |
| validation | 1 (5.0) |  |  |  |
| *data and measurement capability* | *10* | *13* | *21* | *2* |
| validation |  | 1 (7.7) |  |  |
| process mining |  | 1 (7.7) | 2 (9.5) |  |
| advanced computing |  | 1 (7.7) |  |  |
| artificial intelligence |  | 1 (7.7) | 1 (4.8) |  |
| automatic data exchange |  | 1 (7.7) | 1 (4.8) |  |
| computational models | 2 (20.0) | 1 (7.7) |  |  |
| data capability | 2 (20.0) | 1 (7.7) |  |  |
| data exchange |  | 1 (7.7) |  | 1 (50.0) |
| data interpretation |  | 1 (7.7) |  |  |
| machine learning |  | 1 (7.7) | 1 (4.8) |  |
| predictive analytics |  | 1 (7.7) | 3 (14.3) |  |
| Big Data |  | 1 (7.7) | 1 (4.8) |  |
| real-time analysis and reporting |  | 1 (7.7) | 1 (4.8) |  |
| data integration |  |  | 3 (14.3) |  |
| data harmonization and integration |  |  | 2 (9.5) |  |
| data analytics | 1 (10.0) |  | 1 (4.8) |  |
| data quality and access |  |  | 1 (4.8) |  |
| data structure | 1 (10.0) |  | 1 (4.8) | 1 (50.0) |
| process mining techniques |  |  | 1 (4.8) |  |
| routine measurement |  |  | 1 (4.8) |  |
| health economics evaluation |  |  | 1 (4.8) |  |
| data utility | 3 (30.0) |  |  |  |
| impacts measured | 1 (10.0) |  |  |  |
| *healthcare suitability*  (not further defined) | *6* | *9* | *4* | *1* |
| *simulation* | *4* | *2* | *5* | *6* |
| simulation (not further defined) | 4 (100.0) | 2 (100.0) | 4 (80.0) |  |
| Petri Nets |  |  | 1 (20.0) | 3 (50.0) |
| discrete simulation models |  |  |  | 1 (16.7) |
| Markov model for simulation |  |  |  | 1 (16.7) |
| dynamic simulations |  |  |  | 1 (16.7) |
| *automation and conditionality* | *2* | *2* | *8* | *8* |
| CMMN |  |  |  | 4 (50.0) |
| react ad-hoc to changing case-specific conditions |  |  |  | 2 (25.0) |
| repeatable tasks |  |  |  | 1 (12.5) |
| automated and conditional task execution | 1 (50.0) |  | 1 (12.5) | 1 (12.5) |
| automation | 1 (50.0) | 1 (50.0) | 1 (12.5) |  |
| user interaction required |  | 1 (50.0) |  |  |
| automatic data exchange |  |  | 1 (12.5) |  |
| automatic decision tasks |  |  | 1 (12.5) |  |
| conditional execution |  |  | 2 (25.0) |  |
| decision support automation |  |  | 2 (25.0) |  |
| *Efficiency*  (not further defined) | *1* | *5* | *1* | *1* |
| *decision-making* | *2* | *1* | *14* | *9* |
| decision model complexity |  |  |  | 2 (22.2) |
| CMMN |  |  |  | 2 (22.2) |
| operational performance analysis |  |  |  | 1 (11.1) |
| hard-coded decisions |  |  |  | 1 (11.1) |
| non-operational decisions in healthcare |  |  |  | 1 (11.1) |
| decision rule | 1 (50.0) |  |  | 1 (11.1) |
| real-time ad-hoc decisions |  |  |  | 1 (11.1) |
| decision-making | 1 (50.0) |  | 8 (57.1) |  |
| decision support automation |  |  | 4 (28.6) |  |
| integrating decision-making |  | 1 (100.0) | 1 (7.1) |  |
| automatic decision tasks |  |  | 1 (7.1) |  |
| *tools* | *1* | *0* | *19* | *3* |
| complementary tools | 1 (100.0) |  | 13 (68.4) | 3 (100.0) |
| tool development |  |  | 2 (10.5) |  |
| process applications |  |  | 1 (5.3) |  |
| application of tools |  |  | 1 (5.3) |  |
| process mapping techniques |  |  | 1 (5.3) |  |
| combined process and decision modeling tool |  |  | 1 (5.3) |  |
| *information management* | *0* | *2* | *7* | *5* |
| nformation management (not further defined) |  |  | 4 (57.1) |  |
| nowledge management |  |  | 2 (28.6) |  |
| knowledge creation |  | 1 (50.0) | 1 (14.3) |  |
| knowledge modelling |  |  |  | 5 (100.0) |
| Data = extraction counts (%) |  |  |  |  |
